# Derangement of cell cycle markers in peripheral blood mononuclear cells of asthmatic patients as a reliable biomarker for asthma control

**DOI:** 10.1038/s41598-021-91087-5

**Published:** 2021-06-04

**Authors:** Mahmood Yaseen Hachim, Noha Mousaad Elemam, Rakhee K. Ramakrishnan, Laila Salameh, Ronald Olivenstein, Ibrahim Yaseen Hachim, Thenmozhi Venkatachalam, Bassam Mahboub, Saba Al Heialy, Qutayba Hamid, Rifat Hamoudi

**Affiliations:** 1 College of Medicine, Mohammed Bin Rashid University of Medicine and Health Sciences, Dubai, United Arab Emirates; 2 Center for Genomic Discovery, Mohammed Bin Rashid University of Medicine and Health Sciences, Dubai, United Arab Emirates; 3 Sharjah Institute for Medical Research, College of Medicine, University of Sharjah, Sharjah, United Arab Emirates; 4 Meakins-Christie Laboratories, McGill University, Montreal, QC Canada; 5 Division of Surgery and Interventional Science, UCL, London, UK

**Keywords:** Immunology, Molecular biology, Systems biology

## Abstract

In asthma, most of the identified biomarkers pertain to the Th2 phenotype and no known biomarkers have been verified for severe asthmatics. Therefore, identifying biomarkers using the integrative phenotype-genotype approach in severe asthma is needed. The study aims to identify novel biomarkers as genes or pathways representing the core drivers in asthma development, progression to the severe form, resistance to therapy, and tissue remodeling regardless of the sample cells or tissues examined. Comprehensive reanalysis of publicly available transcriptomic data that later was validated in vitro, and locally recruited patients were used to decipher the molecular basis of asthma. Our in-silicoanalysis revealed a total of 10 genes (GPRC5A, SFN, ABCA1, KRT8, TOP2A, SERPINE1, ANLN, MKI67, NEK2, and RRM2) related to cell cycle and proliferation to be deranged in the severe asthmatic bronchial epithelium and fibroblasts compared to their healthy counterparts. In vitro, RT qPCR results showed that (SERPINE1 and RRM2) were upregulated in severe asthmatic bronchial epithelium and fibroblasts, (SFN, ABCA1, TOP2A, SERPINE1, MKI67, and NEK2) were upregulated in asthmatic bronchial epithelium while (GPRC5A and KRT8) were upregulated only in asthmatic bronchial fibroblasts. Furthermore, MKI76, RRM2, and TOP2A were upregulated in Th2 high epithelium while GPRC5A, SFN, ABCA1 were upregulated in the blood of asthmatic patients. SFN, ABCA1 were higher, while MKI67 was lower in severe asthmatic with wheeze compared to nonasthmatics with wheezes. SERPINE1 and GPRC5A were downregulated in the blood of eosinophilic asthmatics, while RRM2 was upregulated in an acute attack of asthma. Validation of the gene expression in PBMC of locally recruited asthma patients showed that SERPINE1, GPRC5A, SFN, ABCA1, MKI67, and RRM2 were downregulated in severe uncontrolled asthma. We have identified a set of biologically crucial genes to the homeostasis of the lung and in asthma development and progression. This study can help us further understand the complex interplay between the transcriptomic data and the external factors which may deviate our understanding of asthma heterogeneity.

## Introduction

Identifying novel markers that can help in the patient classification, clinical outcome prediction, and therapy response was and will remain the ultimate goal for many research projects ^1, 2^. These markers should meet precise specifications and criteria such as reliability, ease of collection and measurement, and non-invasive. Using reliable biomarkers to predict asthma patients’ response to steroid therapy early in the disease course is particularly important and essential to have an optimal response and avoid undesirable side effects ^3^. Using such biomarkers is not routinely requested in severe asthma ^4^ as the biomarkers most extensively studied at present in asthma are Th2 related biomarkers ^5^. Peripheral blood mononuclear cells (PBMCs) (which include lymphocytes, monocytes, and dendritic cells) constitute an essential source of mediators of an allergic response in asthma and serve as an excellent alternative to the costly and challenging approach of obtaining airways samples in severe asthma ^6^. The integrative phenotype–genotype approach is a novel, simple, and powerful tool to identify clinically relevant potential biomarkers. We recently used this approach to identify clinically essential biomarkers in complex and heterogeneous diseases like diabetes ^7^.

Among the omics, bronchial epithelium transcriptomics-driven phenotyping of asthmatic patients showed the potential to discover gene expression profiles characteristic in asthma ^8^. Bronchial epithelium transcriptomics can identify different molecular mechanisms underlying divergent asthmatic phenotypes and have the power to identify novel clinically efficient biomarkers ^9, 10^. It is especially crucial in comparing different groups of adult-onset severe asthma where conventional single gene expression may not capture underlying pathways^11^. Transcriptomics, in comparison to genomics, are more dynamic in terms of changes in response to the environment, treatment, age and is cell-specific, which is very important to understand active heterogeneous diseases like asthma and help in its “prediction” and “diagnosis” ^12^.

There was no link between the identified transcriptomic data and clinical findings in some well-designed transcriptomic analyses in asthma samples, indicating a missing biological relevance if those studies are taken separately^13^. At present, this lost biological relevance can be identified if more comprehensive designs and data analysis are tried^14^. This relatively expensive approach can be more informative if a large number of samples are combined to extract meaningful information by using a large number of datasets available in public databases ^15^. However, such an approach in a broader range of patients has not yet been performed ^16^.

Current asthma research aims to identify novel biomarkers as genes or pathways representing the core drivers in asthma development, progression to the severe form, resistance to therapy, and tissue remodeling. We hypothesized that such genes and pathways should be altered or deranged in lung resident cells and infiltrating immune cells of asthmatics when compared to healthy control to be selected as a novel biomarker. Our research team conducted a comprehensive extensive data analysis from the publicly available transcriptomic datasets to achieve this aim using stringent in-house bioinformatics workflow to identify confounding biological and nonbiological factors that might interfere with the proper identification of such biomarkers. Extensive in silico, in vitro, and in vivo validation of the diagnostic value of the identified genes was followed to explore their translational potentials.

Our research team identified ten genes that were deranged in the severe asthmatic bronchial epithelium and fibroblasts related to cell cycle, cell proliferation, and survival-related pathways^17^. Therefore, further exploration of these genes was done in silico on larger datasets, in vitro on primary cell lines from healthy controls and asthmatic patients, and in clinical samples from locally recruited patients.

## Materials and methods

### In silico exploration of lung and blood transcriptomics dataset

Publicly available transcriptomics datasets of the bronchial epithelium (GSE67472, GSE64913, and GSE76227), bronchial fibroblasts (GSE27335), whole blood, and PBMCs (GSE137394, GSE123750, GSE115823, GSE69683, GSE31773, GSE16032, and GSE73482) from asthmatic patients versus healthy subjects in different settings (n = 1780 sample) were explored to extract the normalized gene expression of the identified genes, as shown in Supplement Table S1 and Fig. S1.

### Genome-wide association studies (GWASs)

We explored the NHGRI-EBI GWAS Catalog (https://www.ebi.ac.uk/gwas/) (v1.0) of published genome-wide association studies ^18^. The file (gwas_catalog_v1.0-associations_e96_r2019-07-12) was downloaded, and we searched for SNPs associated with the strongest Asthma-related SNP-risk allele.

### Methylomes

DNA methylation dataset (GSE85568) of primary airway epithelial cells from 74 asthmatic and 41 nonasthmatic adults was used to investigate the differential methylation of genes of interest between the two groups. Another dataset was explored to analyze the DNA methylation profiles in peripheral blood monocytes (GSE59339) from eosinophilic asthma (n = 21), paucigranulocytic asthma (n = 22), neutrophilic asthma (n = 9), and healthy controls (n = 10) for the same purpose. Normalized methylation scores were extracted from the datasets; accordingly, results are shown in the Supplement (S2).

### Prediction of the specificity of the identified genes to lung cells

The lung gene expression analysis (LGEA) integrative web portal (https://research.cchmc.org/pbge/lunggens/mainportal.html) was used to check the specificity of these shortlisted genes to the different components of the lung ^19^.

### In vivo validation

#### Patient population

From January 2017 to May 2019, individuals were recruited consecutively from the Asthma Clinic in Rashid Hospital, Pulmonary Medicine Department. Thirty-two asthmatic patients were included, 19 were nonsevere asthmatic patients (mild to moderate), and 13 were severe asthmatic subjects (based on the criteria for asthma as per American Thoracic Society). These patients were compared to 12 nonasthmatic volunteer subjects who had no recent infection of the respiratory tract and no histories of allergy or asthma. Participants completed the Asthma Control Test self-assessment. The Ethics Committee of Dubai Health Authority and the University of Sharjah approved the study with REC (Research Ethics Committee) approval number DSREC-11/2017_04, and each subject gave written informed consent after a thorough explanation by the treating physician and the researchers. This study was conducted following the Declaration of Helsinki. The demographic characteristics of the asthmatic patients and control subjects are shown in Supplementary Table S2. Blood was collected, as previously described ^20^.

#### Blood collection protocol and plasma isolation

Twelve milliliters of whole blood were collected from each patient in EDTA-containing blood collection tubes (3 ml each) and transferred immediately within 2 h to Sharjah Institute for Medical Research (SIMR), Sharjah for further processing to isolate PBMCs as previously described ^20^. Twelve milliliters of Histopaque-1077 (Sigma, #10771, Germany) were added to a 50 ml centrifuge tube and brought to room temperature, then 12 ml of whole blood were carefully layered on top of the Histopaque and centrifuged at 400 × *g* for precisely 30 min at room temperature. After centrifugation, the plasma layer and the buffy layer interface were carefully collected with separate Pasteur pipettes and transferred to clean 15 ml conical centrifuge tubes separately, to be frozen at − 80 °C till future use.

### In vitro validation

#### Primary cell lines

Primary cells from healthy and asthmatic patients were isolated from bronchial biopsies in Meakins-Christie Laboratories, The Centre for Respiratory Research at McGill University, and the Research Institute of McGill University Health Centre as previously described ^21^. We compared healthy bronchial epithelium (NHLE, n = 3) to asthmatic cells (DHLE, n = 3), and healthy lung fibroblasts (NHLF, n = 3) were compared to asthmatic lung fibroblasts (DHLF, n = 3). Epithelial cells were revived and maintained using epithelial growth medium PneumaCul-Ex Medium (Stem Cell Technology, Canada), supplemented with 100 units/ml penicillin/streptomycin (Gibco, USA). Primary fibroblasts were maintained in complete Dulbecco’s Modified Eagle’s medium (DMEM) (Sigma-Aldrich, Germany) with 10% fetal bovine serum (FBS) (Sigma-Aldrich, Germany) supplemented with 100 units/ml penicillin/streptomycin (Gibco, USA).

#### mRNA gene expression using qRT-PCR

RNA was extracted using RNAeasy mini kit (Qiagen, Germany) as per the manufacturer’s instructions. RNA purity (OD260/OD280) and quantity were measured using Nanodrop 2000 (ThermoFisher Scientific, USA). The purified RNA was reverse transcribed into cDNA using High-Capacity cDNA Reverse Transcription (Applied Biosystems, USA) as per the manufacturer’s instructions. 5X Hot FIREPol EvaGreen qPCR Supermix (Solis BioDyne, Estonia) was used to quantify mRNA expression of the selected genes using QuantStudio3 (Applied Biosystems, USA). Details of the used primers are in the Supplementary Table S3.

### Statistical analysis

GraphPad Prism version 7.00 for Windows (GraphPad Software, La Jolla California USA) was used for statistical analysis. First, the D’Agostino–Pearson normality test was used to determine whether to perform parametric or nonparametric tests. A one-way ANOVA test was performed to determine whether there were any statistically significant differences between the mean values of the controls and different asthma groups for the gene expression. Prism was used to compute a multiplicity-adjusted P-value for each comparison when ANOVA was used. The same software was used to examine the correlations between the different parameters using linear regression. The student t-test was used to determine the difference between two groups under a given experiment or treatment. A p-value < 0.05 was considered to be statistically significant.

## Results

### ANLN, ABCA1, MKI67, and NEK2 were upregulated in asthmatic epithelial cells but were downregulated in severe asthmatic fibroblasts when compared to healthy controls

**Figure 1:**
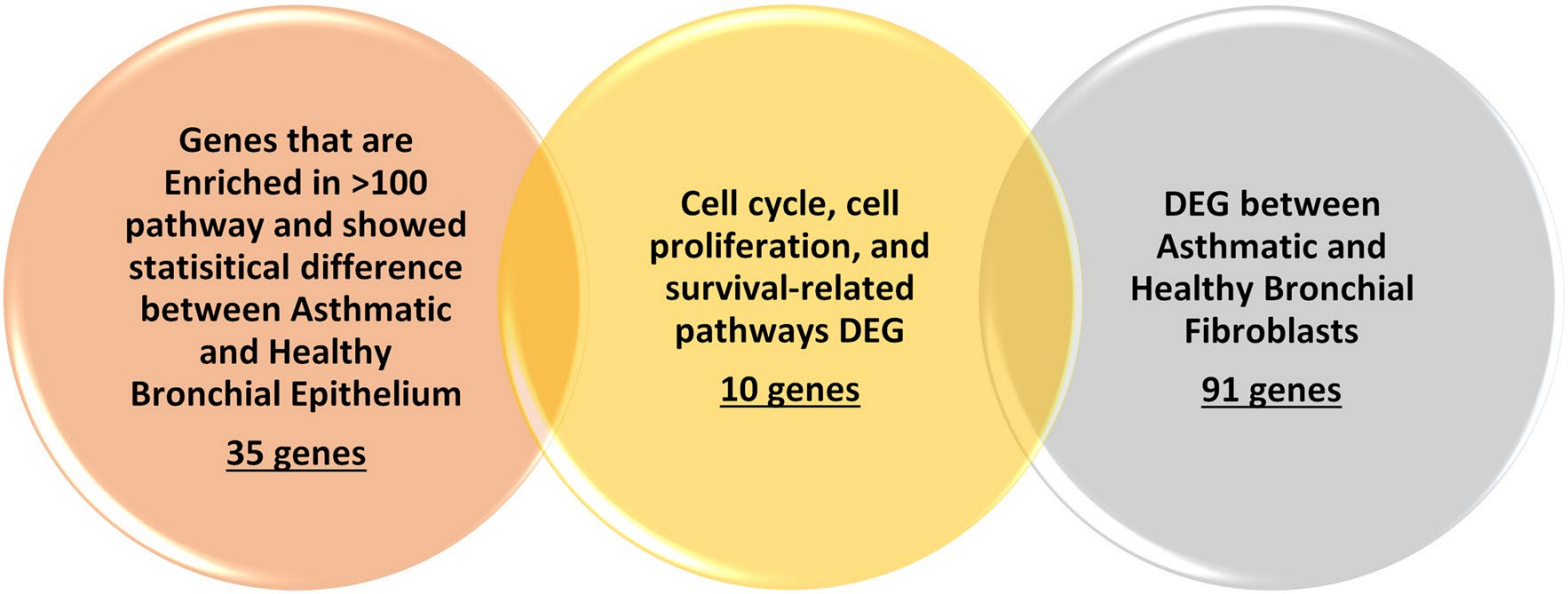
Schematic representation of the filtering of Genes Identified using our bioinformatic pipeline showing the resultant gene list that shares a common pathway mainly related to cell cycle, cell proliferation, and survival.

Our research team identified ten genes that were deranged in the severe asthmatic bronchial epithelium and fibroblasts related to cell cycle, cell proliferation, and survival-related pathways^17^ shown in Fig. 1. Four genes (GPRC5A, SFN, ABCA1, and KRT8) were upregulated in both asthmatic epithelium and fibroblasts, while the rest six genes (TOP2A, SERPINE1, ANLN, MKI67, NEK2, and RRM2) were downregulated in asthmatic fibroblasts despite being upregulated in the asthmatic epithelium. The list of genes and their details are shown in Table 1.

**Table 1:** List of genes related to cell cycle displayed significant differential expression between asthmatic and healthy epithelium in both genders, bronchial brush versus the biopsy sample, and between asthmatic and healthy fibroblasts, from publicly available datasets (GSE64913, GSE76227, and GSE27335).

Genes	Number of enriched pathways	Gender	Biopsy vs brush	In asthmatic epithelium	In asthmatic fibroblasts
ABCA1	117	Both	Both	Upregulated	Upregulated
GPRC5A	108	Both	Both	Upregulated	Upregulated
KRT8	106	Both	Both	Upregulated	Upregulated
SFN	102	Both	Both	Upregulated	Upregulated
TOP2A	175	Both	Both	Upregulated	Downregulated
MKI67	171	Both	Both	Upregulated	Downregulated
RRM2	147	Both	Both	Upregulated	Downregulated
ANLN	109	Both	Both	Upregulated	Downregulated
NEK2	108	Both	Both	Upregulated	Downregulated
SERPINE1	103	Both	Both	Upregulated	Downregulated

**Figure 2:**
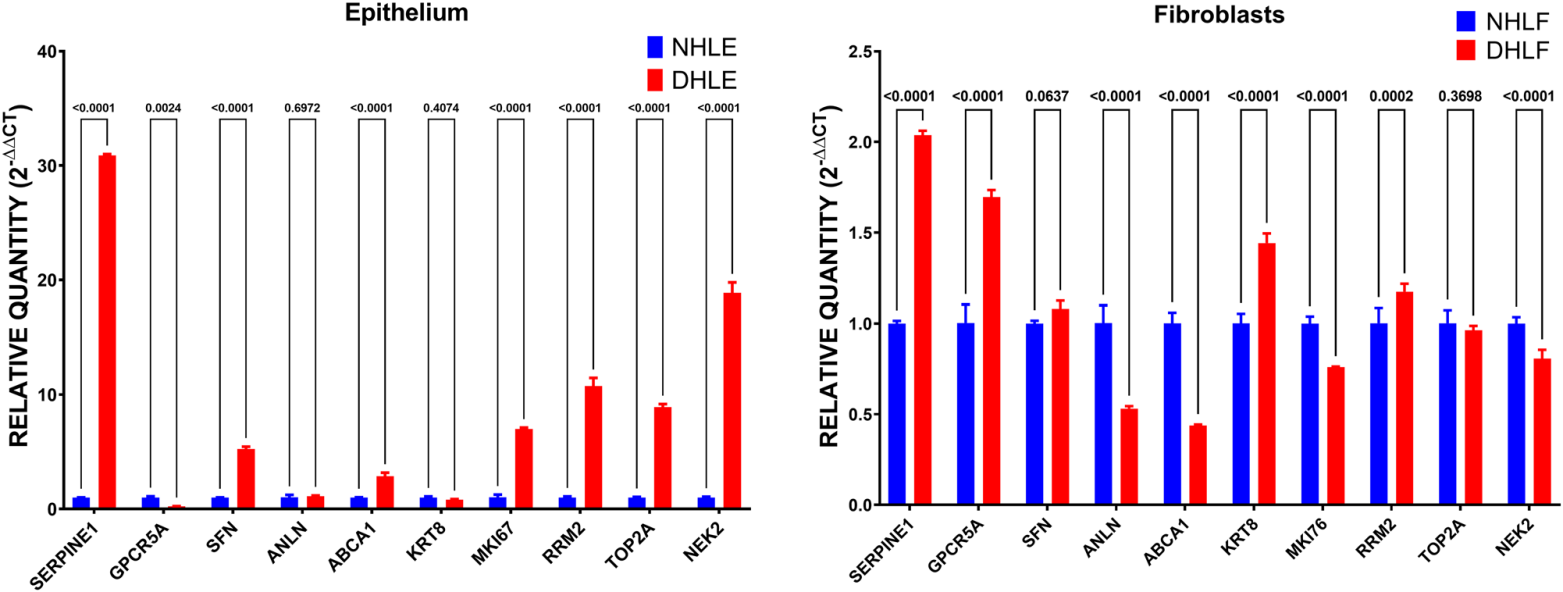
mRNA expression of cell cycle-related genes in healthy bronchial epithelium (NHLE, n = 3) versus asthmatic cells (DHLE, n = 3), and healthy lung fibroblasts (NHLF, n = 3) compared to asthmatic lung fibroblasts (DHLF, n = 3).

The expression of the ten genes was examined on representative structural cells from healthy controls versus asthmatic patients to validate the preliminary bioinformatics findings. mRNA expression of (SERPINE1, GPRC5A, SFN, ANLN, ABCA1, KRT8, MKI67, RRM2, TOP2A, and NEK2) was examined in healthy bronchial epithelium (NHLE, n = 3) versus asthmatic cells (DHLE, n = 3), and healthy lung fibroblasts (NHLF, n = 3) compared to asthmatic lung fibroblasts (DHLF, n = 3) as shown in Fig. 2.

SERPINE1, SFN, ABCA1, MKI67, RRM2, TOP2A, and NEK2 were significantly upregulated in asthmatic epithelium compared to healthy ones; these results match the bioinformatics data while GPRC5A was decreased considerably in asthmatic epithelium compared to a healthy one (p < 0.05). On the other hand, asthmatic fibroblasts showed higher expression of (SERPINE1, GPRC5A, KRT8, and RRM2) and lower (ANLN, ABCA1, MKI67, and NEK2) compared to healthy.

### MKI67, RRM2, and TOP2A showed higher expression in Th2-high asthmatics

**Figure 3:**
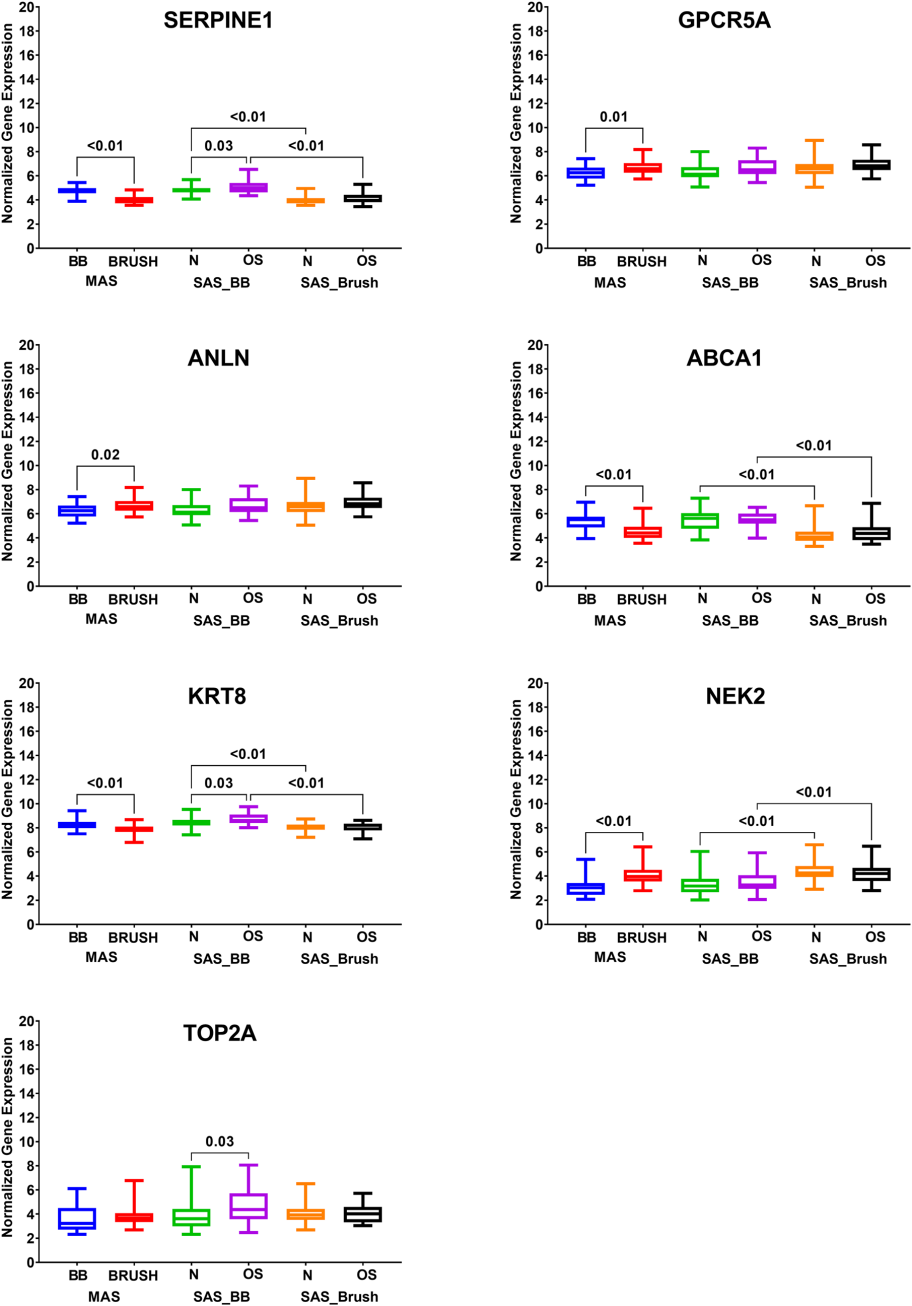
mRNA expression of the ten genes in bronchial epithelium using, GSE76227 transcriptomic dataset that contains the expression data of 190 bronchial biopsies (BB) and epithelial brushing (BRUSH) from Unbiased BIOmarkers in Prediction of REspiratory Disease outcomes (U-BIOPRED) Project. The normalized gene expression of each of the identified genes was extracted and compared between different subgroups. The datasets were subdivided into nonsevere asthmatic (MAS), severe asthmatics (SAS) (oral steroid naïve (N) vs. oral steroid users (OS).

Asthmatics patients can be categorized according to the levels of type 2 inflammation into ‘T helper 2 (Th2)-high’ and ‘Th2-low’ ^22^. The type 2 immune response-driven endotype consists of T2-high asthma endotype encompasses several related subtypes in both children and adults^23^. At the same time, T2-low endotype included neutrophilic or paucigranulocytic airway inflammation with greater resistance to steroids^24^. The differential expression for the identified genes between Th2-high and Th2-low asthma phenotypes was investigated using the GSE67472 dataset, which includes the airway epithelial brushings transcriptomics data of 62 subjects with mild-to-moderate asthma not on inhaled steroids and 43 healthy controls. Asthma subjects were stratified into two subgroups, Th2-high, and Th2-low asthma, based on their expression of a three-gene signature of Type 2 inflammation: POSTN, SERPINB2, and CLCA1. Interestingly, our analysis showed that MKI67, RRM2, and TOP2A expression in Th2-high asthmatics were higher than controls, as shown in Fig. 3. On the other hand, MKI67 and TOP2A were higher in Th2-high compared to Th2-low asthmatics. Interestingly, TOP2A showed lower expression in Th2-low asthmatics when compared to healthy controls too.

### Oral steroids upregulated the SERPINE1 and KRT8 mRNA expression in the bronchial biopsy from severe asthmatics but not in the bronchial brush tissue

The mRNA expression of the identified genes can indicate the difference in the cells of the given sample examined in terms of pure epithelial cells or mixed mesenchymal and/or infiltrating immune cells; this necessitates the examination of such genes in biopsies of lung rather than just bronchial brushes. To examine that transcriptomic dataset (GSE76227) was used. This dataset contains the expression data of 190 bronchial biopsies and epithelial brushing from Unbiased BIOmarkers in Prediction of REspiratory Disease outcomes (U-BIOPRED) Project. The normalized gene expression of each of the identified genes was extracted and compared between different subgroups. The datasets were subdivided into nonsevere asthmatic (bronchial brush vs. biopsy), severe asthmatics (oral steroid naïve (bronchial brush vs. biopsy) vs. oral steroid users (bronchial brush vs. biopsy)).

**Figure 4:**
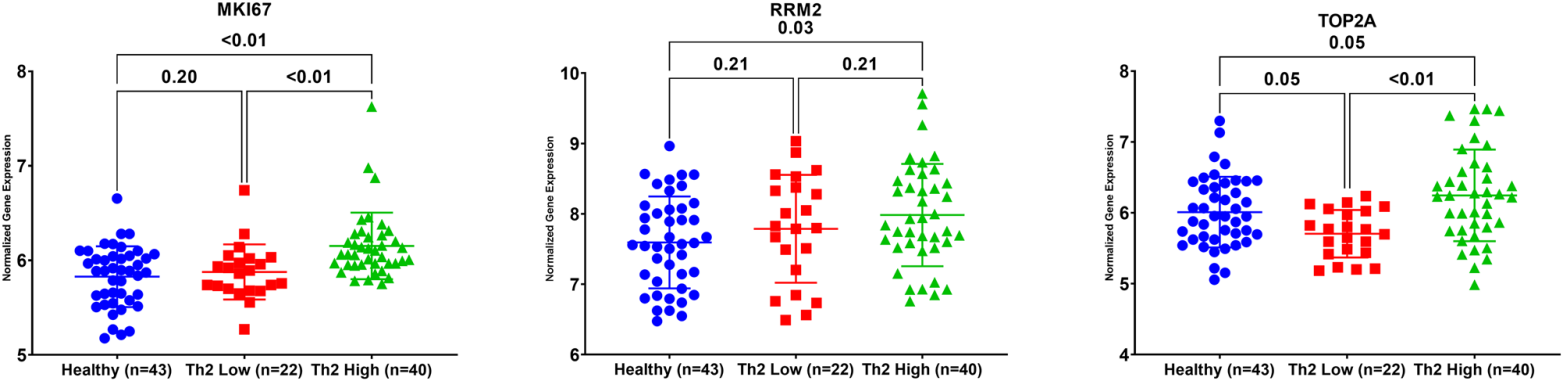
mRNA expression of (MKI67, RRM2, and TOP2A) genes in bronchial epithelium using GSE67472 dataset to compare healthy controls (n = 43) to Th2-high asthmatics (n = 40) and Th2 low asthmatics (n = 22).

As shown in Fig. 4, SERPINE1, ABCA1, and KRT8 showed higher expression in bronchial biopsies than the pure bronchial epithelium, in moderate and severe asthma, with and without oral steroid use. Oral steroids upregulated the SERPINE1, and KRT8 mRNA expression in the bronchial biopsy from severe asthmatics but not in the bronchial brush tissue indicating the source of their upregulation might be from stromal cells rather than epithelial cells in response to steroids. Another possible source of this increase can be redistributing T cells away from the epithelium^25^. Steroids did not affect the mRNA expression of ABCA1 in both types of samples in severe asthmatics.

GPCR5A and ANLN showed higher expression in nonsevere bronchial epithelium than bronchial biopsy, while NEK2 showed higher expression in nonsevere and severe bronchial epithelium compared to bronchial biopsy. Interestingly, TOP2A showed upregulation in bronchial biopsies of asthmatics who were on oral steroids only.

### Summary of in silico and in vitro analysis of the ten genes in bronchial epithelium and fibroblasts of asthmatic patients

The summary of the selected genes mRNA expression in the lung showed that in vitro RT qPCR results confirmed the in-silico findings for most of the genes identified. The Lung Gene Expression Analysis (LGEA) showed that KRT8, GPRC5A, and SFN have the highest specificity to bronchial epithelial cells exclusively. While RRM2, TOP2A, ANLN, MKi67, and NEK2 showed the highest correlation to proliferative mesenchymal progenitors. NEK2 was expressed by many cell types, while SERPINE1 was specific to pericytes, and ABCA1 was specific to immune/myeloid cells. The summary of the ten genes in silico and in vitro results in the lung are listed in Table 2.

**Table 2:**
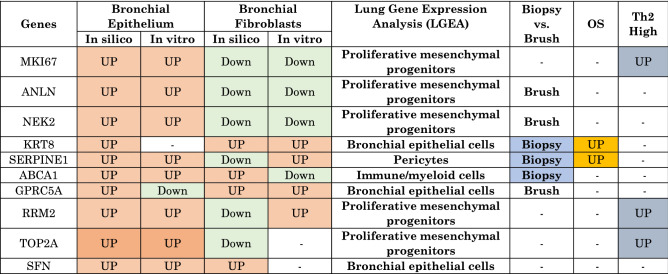
Summary of in silico and in vitro analysis of the ten genes in bronchial epithelium and fibroblasts of asthmatic patients.

If their expression is specific to bronchial brush or biopsy or not, their response to oral steroids (OS) and their expression level in Th2 high versus Th2 low asthma.

MKI67, ANLN, and NEK2 were upregulated in asthmatic bronchial epithelium while they were downregulated in asthmatic bronchial fibroblasts, although according to LGEA, those genes are specific proliferative mesenchymal progenitors who might indicate the loss of this characteristic in severe asthmatic fibroblasts. Among the three, MKI67 was shown to be upregulated in Th2 High asthmatic epithelium along with RRM2 and TOP2A, which might be an EMT marker in asthmatic epithelium too.

### Protein’s products of the identified genes are specific to NK and T regulatory cells

After elucidating the role of the ten genes in the lung, the next question was whether these genes are altered in the circulating immune cells to check their feasibility as a biomarker in asthmatic patients. Human Protein Atlas database was explored https://www.proteinatlas.org/, to validate if the identified genes can be detected as mRNA in the blood or not and what cells express them as detailed in the Table 3. Proteins of two genes were documented to be secreted from cells either to blood (SERPINE1) or other tissues (SFN) and can be detected in the blood. The rest genes’ products were intracellular (MKI67, KRT8, TOP2A, ANLN, and NEK2), intracellular, and membranous (GPRC5A, RRM2, and ABCA1). Some of these proteins were specific to the blood cell lineage of dendritic cells “plasmacytoid DC,” granulocytes, NK-cells (KRT8), granulocytes “eosinophil and basophil,” and for T-cells “naive CD4 T-cell, T-reg” and NK-cells (MKI67). On the other hand, some gene products showed lineage enrichment for basophil (SFN), eosinophil and neutrophil (ABCA1), NK-cell (SERPINE1), and T-reg (RRM2, TOP2A, and ANLN). NEK2 showed Low lineage specificity and low cell type specificity as its mRNA was detected in many blood cells.

**Table 3:** Gene’s products and blood or cell lineage using the Human Protein Atlas database were explored https://www.proteinatlas.org/.

Genes	Secretome annotation	RNA blood cell lineage specificity	RNA blood cell lineage distribution	RNA blood cell type specificity	RNA blood cell type distribution
SFN	Secreted in other tissues	Lineage enriched (granulocytes)	Detected in single	Cell type enriched (basophil)	Detected in single
SERPINE1	Secreted to blood	Lineage enriched (NK-cells)	Detected in single	Cell type enriched (NK-cell)	Detected in single
ANLN	Intracellular	Low lineage specificity	Detected in single	Cell type enhanced (T-reg)	Detected in single
KRT8	Intracellular	Group enriched (dendritic cells, granulocytes, NK-cells)	Detected in many	Cell type enhanced (plasmacytoid DC)	Detected in some
MKI67	Intracellular	Group enriched (T-cells, NK-cells)	Detected in many	Cell type enhanced (naive CD4 T-cell, T-reg)	Detected in some
GPRC5A	Intracellular, Membrane	Lineage enriched (granulocytes)	Detected in single	Cell type enhanced (basophil)	Detected in some
TOP2A	Intracellular	Low lineage specificity	Detected in many	Cell type enhanced (T-reg)	Detected in some
ABCA1	Intracellular, Membrane	Lineage enriched (granulocytes)	Detected in many	Group enriched (eosinophil, neutrophil)	Detected in many
RRM2	Intracellular, Membrane	Lineage enriched (T-cells)	Detected in many	Cell type enhanced (T-reg)	Detected in many
NEK2	Intracellular	Low lineage specificity	Detected in many	Low cell type specificity	Detected in many

The generated shortlist genes were differentially expressed in bronchial epithelium and bronchial mesenchymal cells like fibroblasts and were lineage and cell-specific to individual immune cells, so we tried to explore their dynamic expression in the blood of asthmatics under different settings and the PBMCs of locally recruited patients.

### All identified genes were expressed in peripheral blood lymphocytes

GSE31773 dataset was extracted as it showed the comparison of mRNA expression in circulating T-cells from patients with severe asthma compared to healthy controls. All identified genes were expressed in CD4+ and CD8+ PBMCs, indicating their feasibility to serve as potential biomarkers in blood. Next, we explored the mRNA expression level of GPRC5A, SFN, ABCA1, KRT8, TOP2A, SERPINE1, ANLN, MKI67, NEK2, and RRM2 in the blood of asthmatic patients compared to healthy controls.

### NEK2 and GPRC5A mRNA expression were lower in severe asthmatic patients compared to healthy controls

**Figure 5:**
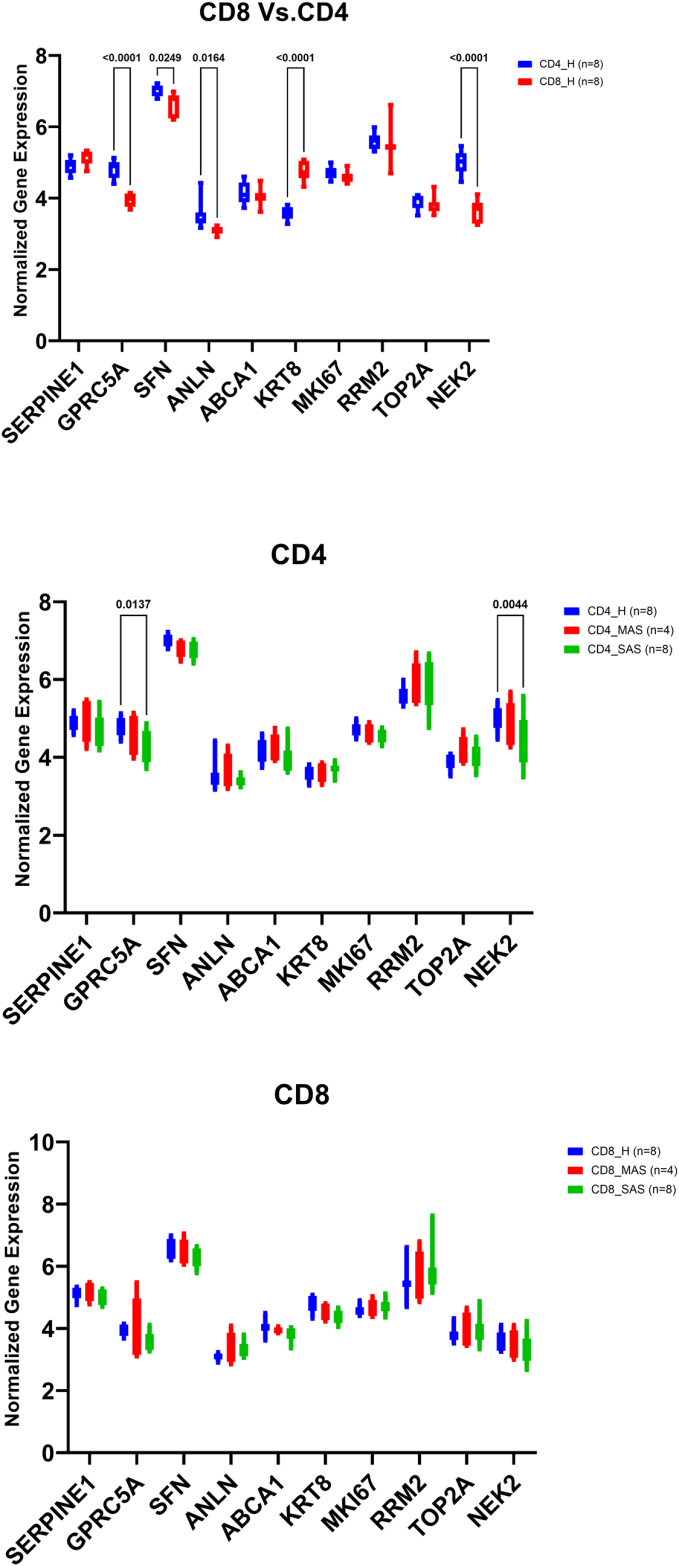
Normalized gene expression of genes identified in circulating T-cells (CD4 versus CD8) of asthmatic patients “MAS = mild to moderate and SAS = severe” compared to healthy (H) controls extracted from the expression profile of publicly available datasets (GSE31773).

The mRNA expression of GPRC5A, SFN, ANLN, and NEK2 was higher in CD4+ T lymphocytes than CD8+ T lymphocytes (p < 0.05). Only KRT8 mRNA expression was higher in CD8+ T lymphocytes in contrast to CD4+ T lymphocytes (p < 0.05). Interestingly, only GPRC5A and NEK2 mRNA expression in CD4+ T lymphocytes were lower in severe asthmatic patients compared to healthy controls (p < 0.05), as shown in Fig. 5.

### GPRC5A, SFN, ABCA1, and NEK2 showed specific and significant differential expression in asthmatic patients’ blood compared to healthy control

**Figure 6:**
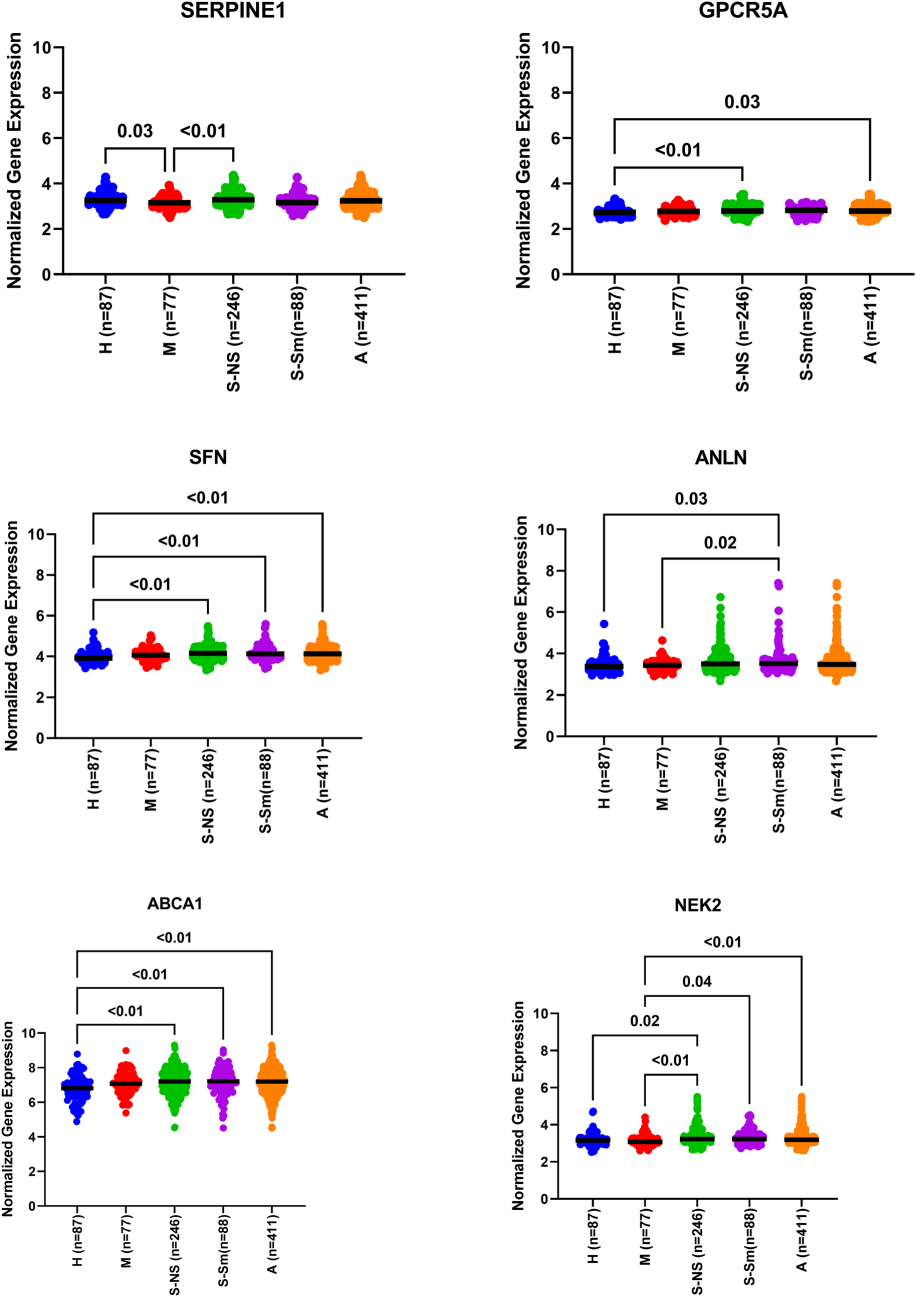
Normalized gene expression of genes identified in the blood of asthmatic patients compared to healthy controls extracted from the expression profile of publicly available datasets (GSE69683) collected in the U-BIOPRED study, where (H) represents the 87 healthy controls, (M) represents the 77-moderate asthma, (S) represents the 246 nonsmoker’s severe asthma, and 88 smoker’s severe asthma, (A) represents the sum of all asthmatic patients in all severities.

The expression profile of the publicly available dataset (GSE69683) of blood transcriptomic from a large number of participants was extracted. A total number of 498 participants divided into 87 healthy controls, 77 moderate asthma, 246 nonsmoker’s severe asthma, and 88 smoker’s severe asthma collected in the U-BIOPRED study, as shown in Fig. 6. Our reanalysis showed that GPRC5A, SFN, ABCA1, and NEK2 mRNA expression was upregulated in asthmatic patients compared to healthy controls (P < 0.05), specifically the nonsmoker severe asthmatics. SERPINE1 mRNA expression was lower in nonsevere asthmatics compared to severe and healthy controls.

### SFN and ABCA1 were higher while MKI67 was lower in severe asthmatic with wheeze compared to nonasthmatics with wheezes

**Figure 7:**
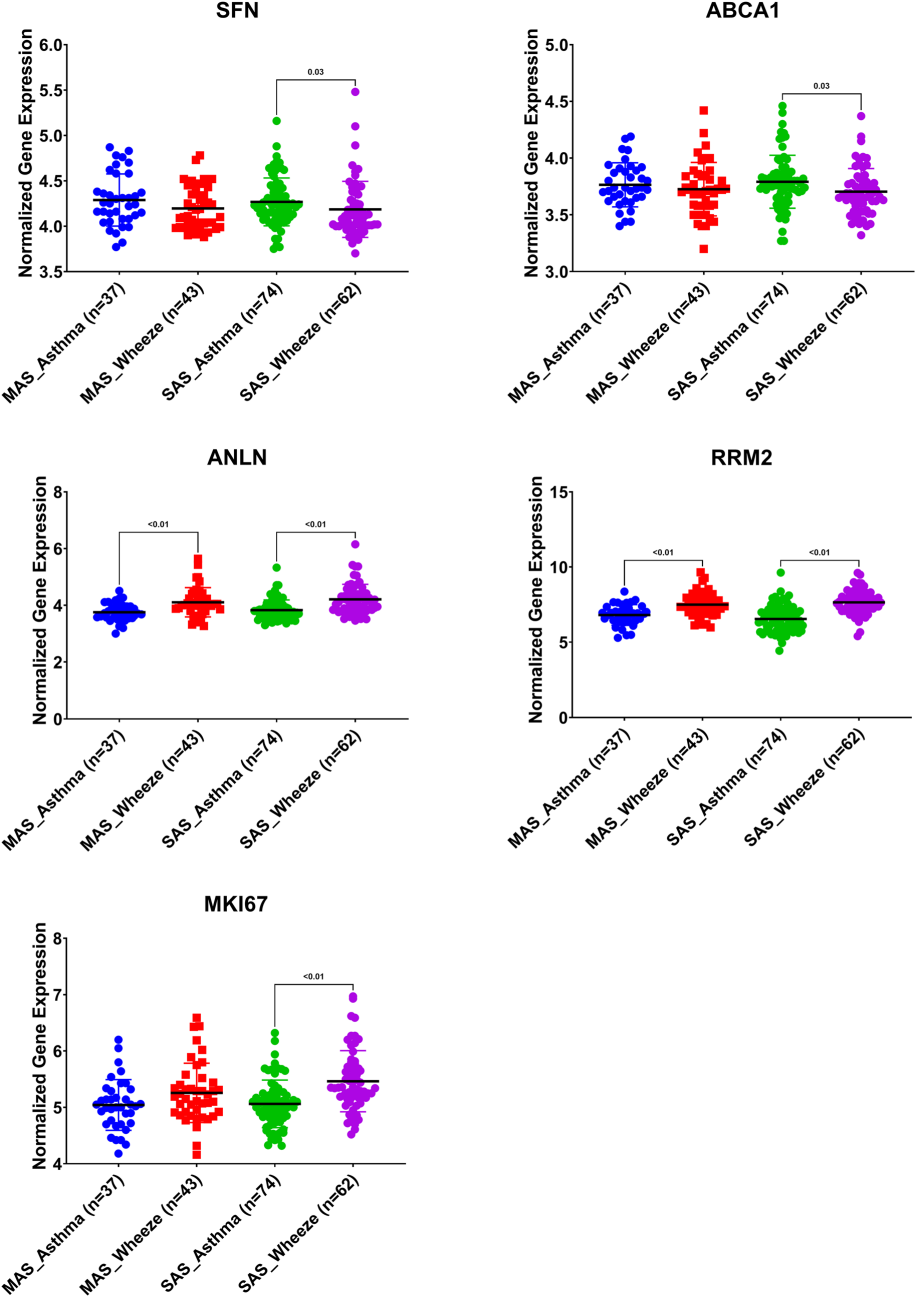
Normalized gene expression of genes identified in whole blood of children with nonsevere and severe wheezes were compared to nonsevere and severe asthmatics extracted from the expression profile of publicly available datasets (GSE123750), where nonsevere asthmatic (MAS_Asthma) and severe asthmatic (SAS_Asthma) patients were compared to patients presented with nonsevere wheezes (MAS_Wheeze) and severe wheezes (SAS_Wheeze).

The correlation of the shortlisted genes to wheeze in asthmatic versus nonasthmatic children was investigated using (GSE123750) dataset. GSE123750 is a cross-sectional study of school-age children with severe asthma or mild, moderate asthma, and pre-school age children with severe wheeze or mild, moderate wheeze. Blood was collected at baseline. mRNA expression of the identified genes in the whole blood of children was compared between the two groups. As shown in Fig. 7, SFN, ABCA1 mRNA expression was significantly higher, while MKI67 mRNA expression was lower in severe asthmatic compared to severe wheezing children. On the other hand, ANLN and RRM2 mRNA expression were significantly lower in asthmatics than nonasthmatic wheezers (both moderate and severe forms).

### House dust mite downregulates SERPINE1 and ABCA1 mRNA expression in CD lymphocytes

**Figure 8:**
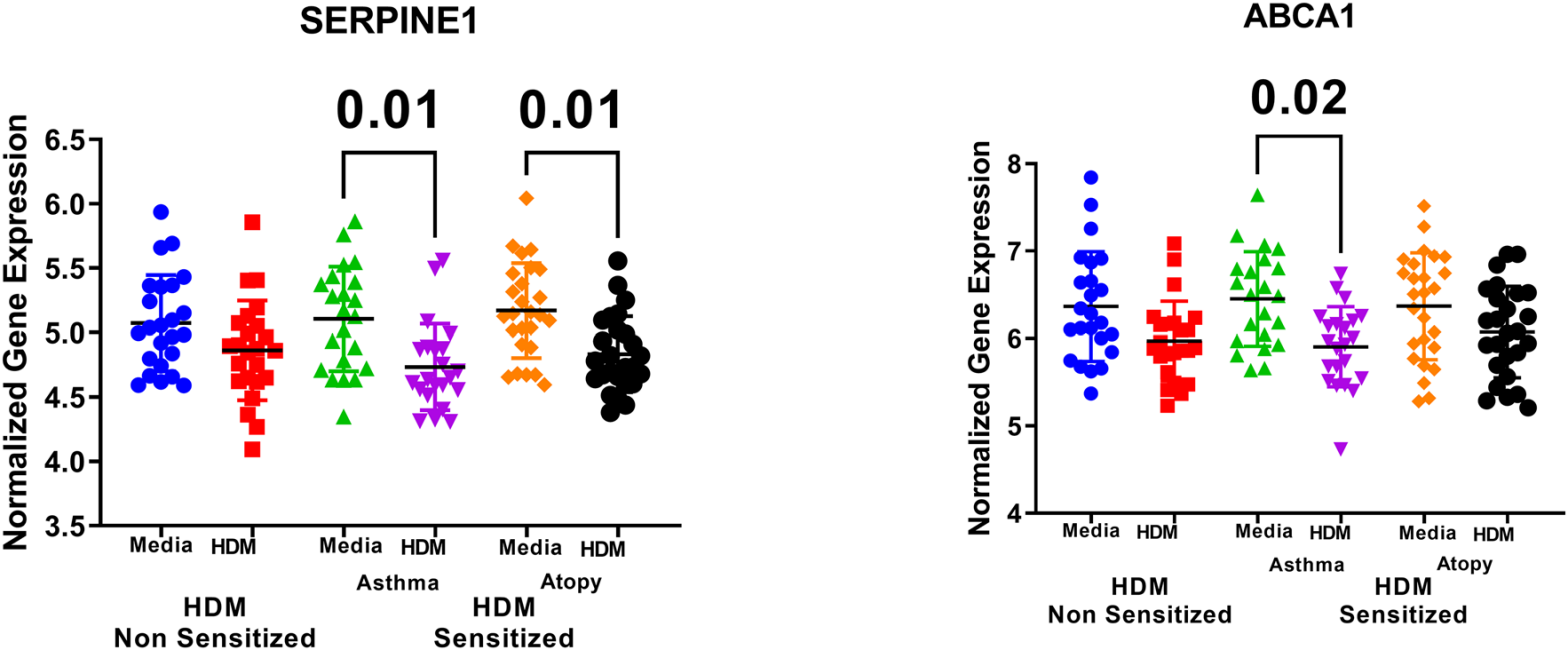
Normalized gene expression of genes identified in CD4 lymphocytes stimulated with HDM compared to media stimulation only in healthy, atopic, and asthmatic patients extracted from the expression profile of publicly available datasets (GSE73482).

HDM is a significant allergen source and a significant cause of allergic rhinitis and allergic asthma ^26^. We explored whether the identified genes were stimulated by HDM or not using GSE73482 transcriptomic dataset. SERPINE1 mRNA expression was significantly downregulated in asthmatic or atopic patients CD4+ T Lymphocytes when challenged with HDM, while ABCA1 was downregulated in asthmatic samples only, as shown in Fig. 8. Our analysis showed that HDM did not alter the gene expression of the other genes identified, indicating that they are related to pathways where HDM had no role.

### Viral respiratory tract infections upregulated KRT8, MKI67, RRM2, TOP2A mRNA expression in asthmatic patients

**Figure 9:**
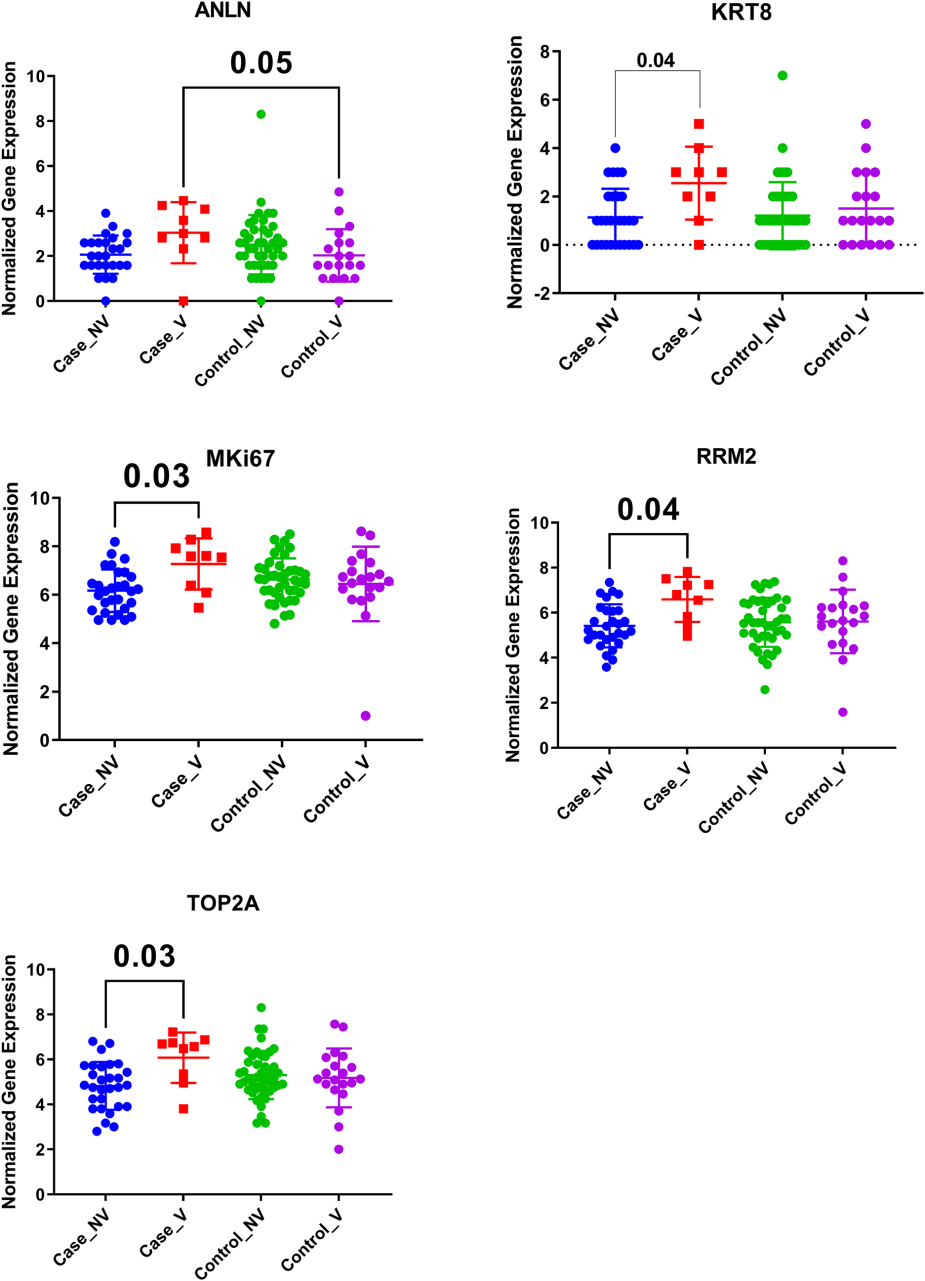
Normalized gene expression of genes identified in blood taken from asthmatic children at baseline (Case_NV) and after they develop viral infections (Case_V) compared to healthy controls (Control_NV and Control_V) extracted from the expression profile of publicly available datasets (GSE115823).

To examine the effect of viral infection on the gene expression of the identified genes, we extracted the normalized gene expression of the GSE115823 transcriptomic dataset where 208 children with exacerbation prone asthma were enrolled with baseline samples collected and were prospectively monitored for the onset of cold symptoms (Events) for six months. KRT8, MKI67, RRM2, and TOP2Agene expression were upregulated significantly in asthmatic who developed cold symptoms, as shown in Fig. 9. ANLN was upregulated in asthmatic who got cold compared to healthy who got colds, indicating its specificity to asthma exacerbation to viral infections.

### PBMCs of asthmatic patients during the exacerbation phase express higher RRM2 compared to the convalescent phase

**Figure 10:**
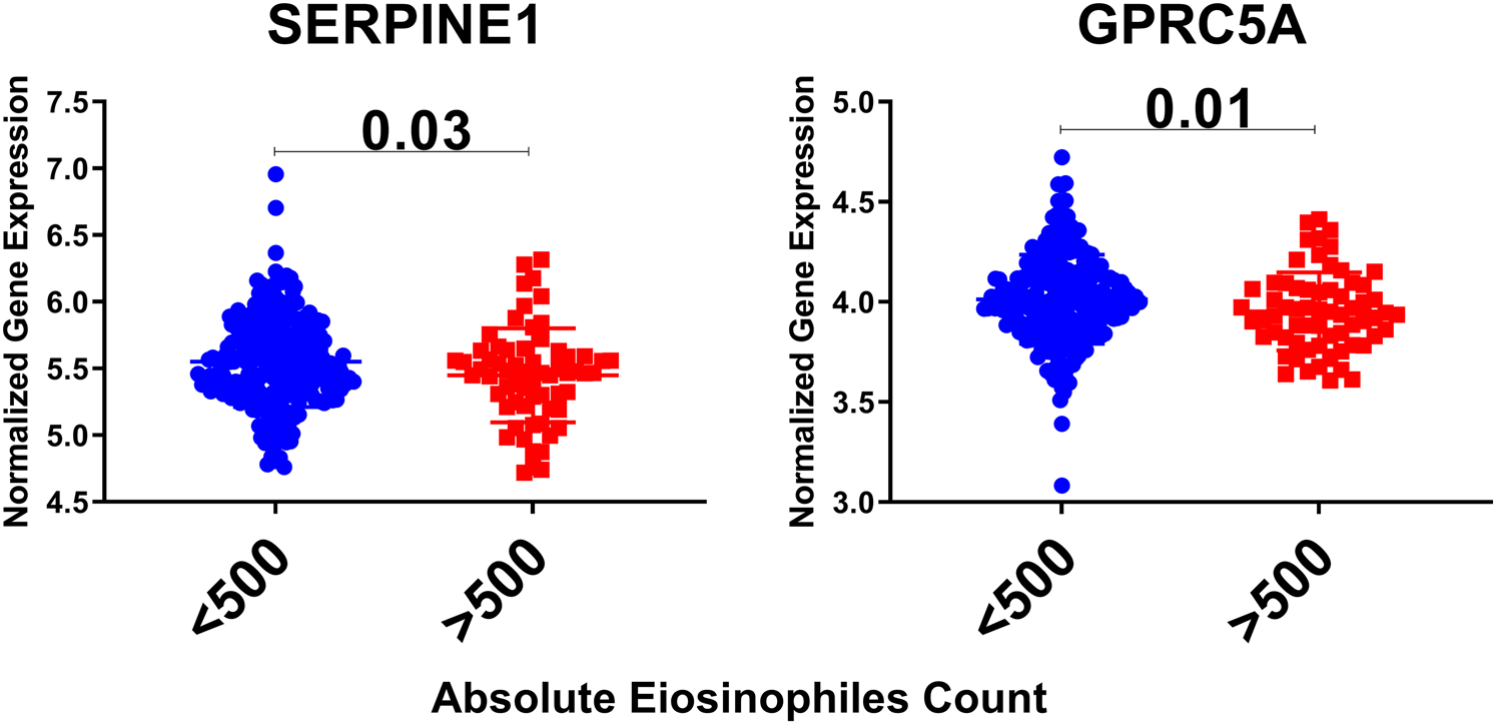
Normalized gene expression of genes identified in PBMCs taken from asthmatic patients during the acute/exacerbation phase and the convalescent phase extracted from the expression profile of publicly available datasets (GSE16032).

GSE16032 dataset was chosen to identify the differential expression of the selected genes in PBMCs taken from asthmatic patients during the acute/exacerbation phase and the convalescent phase, as shown in Fig. 10. Interestingly, only RRM2 was upregulated in the acute/exacerbation phase of asthma and returned to the basal level when the attack subsides.

### SERPINE1 and GPRC5A mRNA expression in whole blood of eosinophilic asthma was significantly lower compared to non-eosinophilic

**Figure 11:**
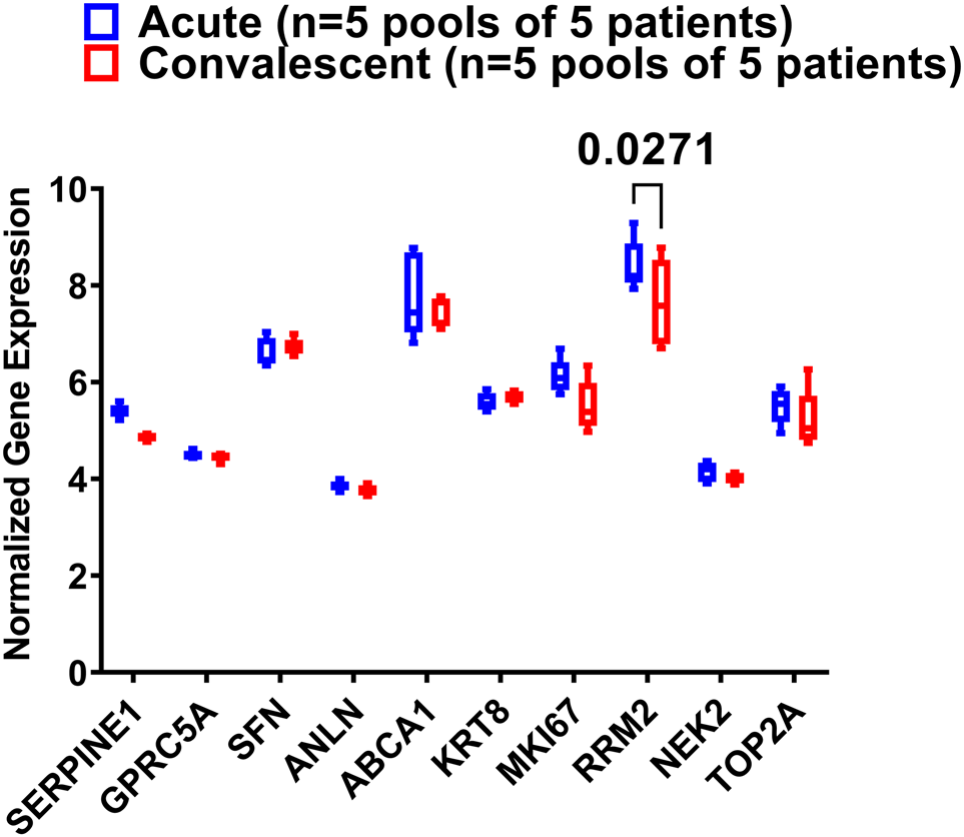
Normalized gene expression of genes Identified in whole blood of eosinophilic asthma (> 500 cells) compared to non-eosinophilic (eosinophils count less than 500) extracted from the expression profile of publicly available datasets (GSE137394).

To explore the correlation of the shortlisted genes to the eosinophilic phenotype of asthma, (GSE137394) dataset was reanalyzed. mRNA expression of the identified genes in whole blood of eosinophilic asthma (> 500 cells) compared to non-eosinophilic (eosinophils count less than 500) were explored, as shown in Fig. 11. Only SERPINE1 and GPRC5A were significantly lower in eosinophilic asthmatics (p = 0.03, and 0.01, respectively). This indicates that SERPINE1 and GPRC5A mRNA levels can identify the eosinophilic subtype of a severe asthma phenotype.

### The ten genes have never been linked to SNPs in asthma

Genome-wide association studies (GWASs) of asthma have identified many risk alleles and loci that can be a potential source for diagnostic biomarkers. NHGRI-EBI GWAS Catalog was explored to identify 1174 SNPs with the strongest Asthma-related SNP-risk allele. Interestingly, none of these SNPs were reported in any of the ten genes identified.

### Methylomes

Another explanation of differential expression of mRNA of the shortlisted genes in asthmatic after excluding genetic alterations in terms of SNPs was epigenetic modifications. DNA methylation dataset (GSE85568) in primary airway epithelial cells from 74 asthmatic and 41 nonasthmatic adults was used to investigate the differential methylation of genes of interest between asthma and healthy controls. There was no significant difference between the methylation status of identified genes in lung epithelium, indicating that epigenetic changes are not responsible for the differential expression seen in severe asthma.

### SERPINE1, ABCA1, and RRM2 were downregulated in severe asthmatics

**Figure 12:**
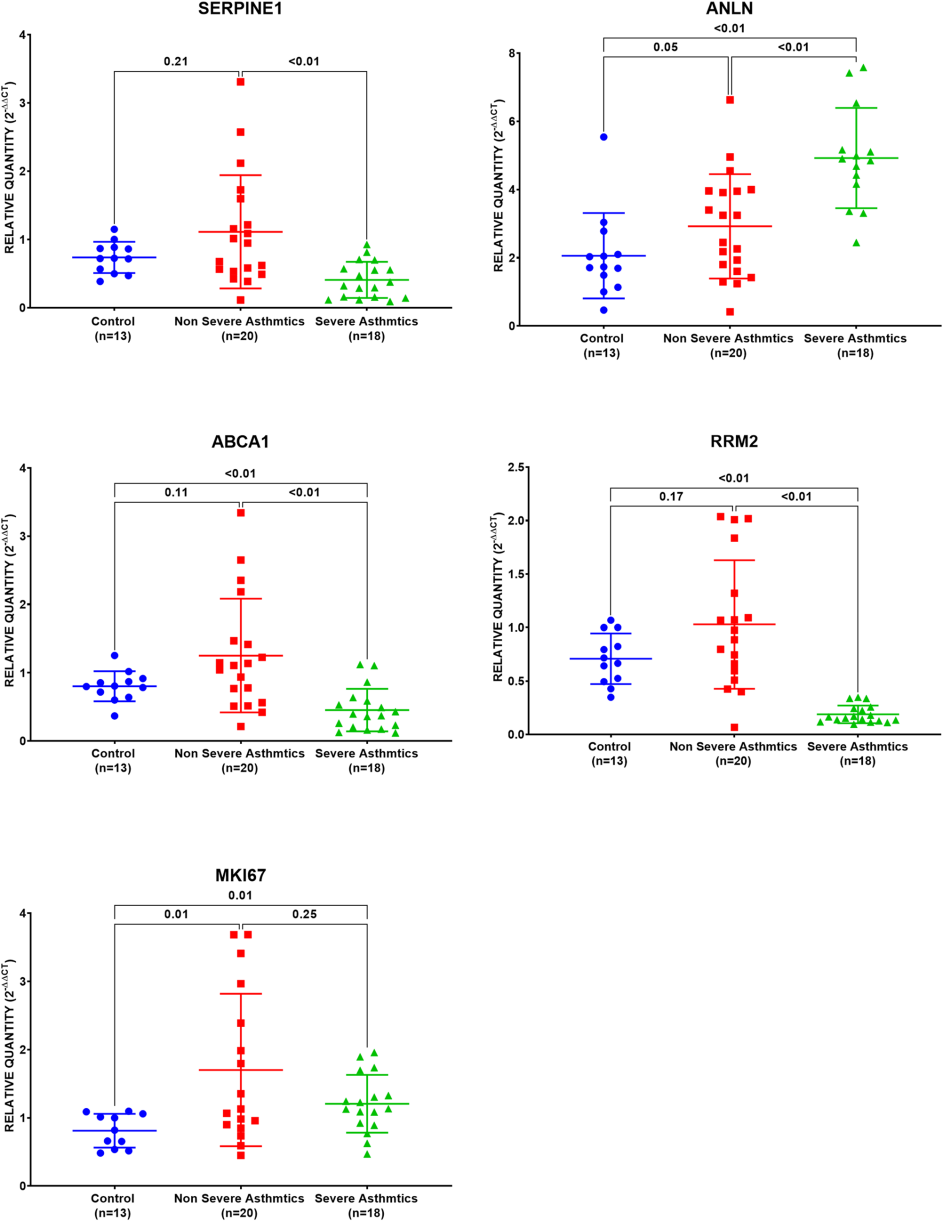
mRNA gene expression using RT qPCR of the ten genes in PBMC of the locally recruited cohort, healthy controls (n = 13), Nonsevere asthmatics (n = 20), and severe asthmatic (n = 18).

mRNA expression of the ten genes was assessed in a locally recruited cohort of asthmatic patients compared to healthy controls; the genes with a significant difference are plotted in Fig. 12. ANLN was the only gene that showed increased expression levels with the severity of the disease. ANLN mRNA expression was higher in asthmatics (nonsevere and severe) and was higher in severe compared to nonsevere asthmatics PBMCs (p < 0.05). The rising level of ANLN with the disease might be used to monitor the disease progression. MKI67 and SERPINE1 expression was higher in nonsevere asthma but was downregulated in severe asthmatics compared to healthy controls (p < 0.05). ABCA1 and RRM2 expression were lower in severe asthmatics than nonsevere asthmatics and healthy controls, making them potential biomarkers to differentiate the asthma severity.

### SERPINE1, GPCR5A, SFN, ABCA1, MKI67, and RRM2 are upregulated in well-controlled asthma

**Figure 13:**
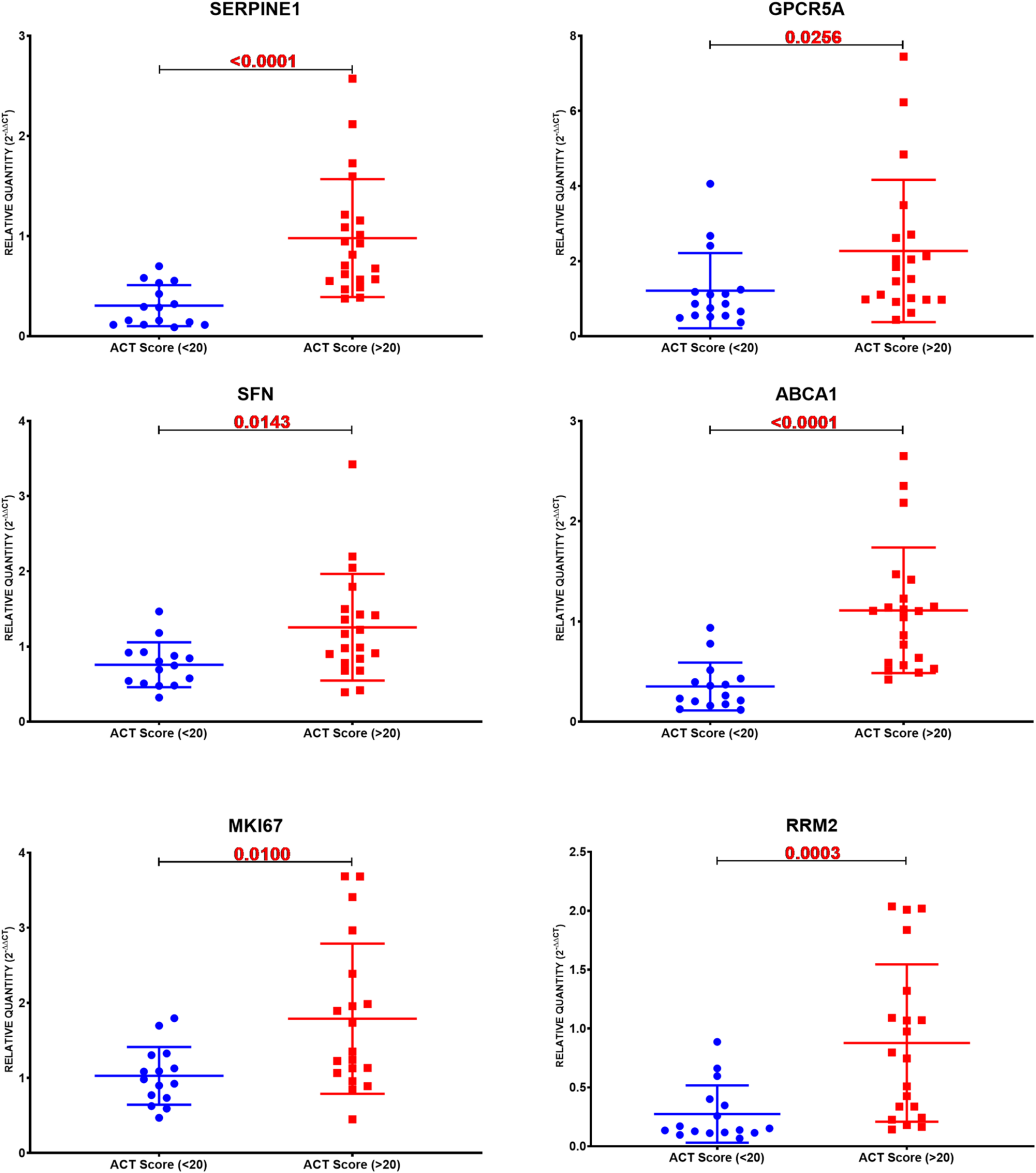
mRNA gene expression using RT qPCR of the ten genes in PBMC of a locally recruited cohort, the asthmatic patients were divided according to the ACT score into two groups: a well-controlled group (ACT > 20) and those with poor control (ACT < 20).

Asthma Control Test (ACT) is a self-administered patient tool for identifying those with poorly controlled asthma. The scores range from 5 (poor control of asthma) to 25 (complete control of asthma), with higher scores reflecting greater asthma control. An ACT score > 20 indicates well-controlled asthma. When we divided our patients accordingly, SERPINE1, GPCR5A, SFN, ABCA1, MKI67, and RRM2 are upregulated in well-controlled asthma, as shown in Fig. 13. This shows that dividing the patients according to ACT, a more objective evaluation of the condition and management compliance, is more accurate to have a homogenous expression of biomarkers in blood samples.

## Discussion

There is currently an imminent need to discover new biomarkers to classify different asthmatic phenotypes, predict their clinical behavior and therapeutic response, and identify new therapeutic targets ^8, 9^. Those markers need to be less invasive and more technically convenient than the currently available ^10^. Despite that necessity, only a few markers are implemented in clinical practice ^11^. The use of transcriptomic-driven biomarker identifications is a promising approach. However, many studies using microarrays showed extreme method-to-method variations in the results of the significant differentially expressed genes (DEG) by using different approaches that seek to shorten the list of identified genes ^27^. These limitations affect the proper identification of truly DEG from the equally expressed genes between disease and control ^28^.

Based on that, some of these successful biomarkers showed significant limitations in different populations and settings due to the lack of a unified system of patients’ classification and lack of complete understanding of confounding comorbidities, co-existing conditions, sampling time, and type besides the treatment history of asthmatic patients enrolled in the biomarker assessment ^12^. For example, atopic status, smoking, and the use of inhaled corticosteroids (ICS) are known confounders for the interpretation of asthma biomarkers ^13^. Another layer of obstacles that block the appropriate utilization of transcriptomic datasets is countless elusive experimental factors called confounding factors that can be biological and nonbiological ^29^. Identification of confounding factors is essential for accurate transcriptomic analysis^30^, but this task is not straightforward as many of these confounding factors are hidden and are difficult to detect before causing systematic bias^31^.

### Cell cycle-related pathways are significantly enriched in the DEG identified

Our research team identified ten genes that were deranged in the severe asthmatic bronchial epithelium and fibroblasts related to cell cycle, cell proliferation, and survival-related pathways^17^. In subjects with severe asthma, there is an increased cellular proliferation in the airway contributing to a thickened epithelium leading to progressive decline in lung function^32^. After a dusty hay challenge in the bronchial epithelium, activation of cell cycle genes shows underlying impaired regeneration of the epithelial barrier in asthmatics ^33^. In severe eosinophilic asthma, fibroblasts can secrete exosomes to control epithelial cell proliferation, further increasing airway remodeling in asthma^34^. Interestingly, such asthmatics myofibroblasts showed reduced proliferative responses but increased cytokine production confirming their inflammatory profile^35^.

Our results showed that cell cycle-related pathways are significantly enriched in the top DEG identified. This finding goes with the previous report where increased cellular proliferation was documented in severe asthmatic airways compared to other phenotypes and healthy control, leading to a progressive decline in lung function and airway remodeling in those patients^32^. Cell cycle-related genes were differentially expressed in different tissues from children with asthma, indicating the importance of such a pathway in disease progress^36^. Upon injury, the bronchial epithelium can secrete inflammatory and fibrogenic cytokines when it shows asynchronous mitosis^37^. The effect of the underlying subepithelial fibroblasts can increase the proliferation of epithelial cells through TGF‐β signaling^38^. Most of these genes that we identified were reported to be related in a way or another to asthma and lung inflammation.

### In lung

Our results showed that MKI67, ANLN, and NEK2 were upregulated in asthmatic bronchial epithelium while they were downregulated in asthmatic bronchial fibroblasts, although according to LGEA, those genes are specific proliferative mesenchymal progenitors who might indicate the loss of proliferative characteristic in severe asthmatic fibroblasts. Of note, their expression was higher in the bronchial brush than biopsy, which might reveal the possible contribution of the epithelium to airway remodeling through epithelial-mesenchymal transition (EMT) expression proliferative mesenchymal progenitors genes^39^. Such EMT is essential in asthma airway remodeling^40^ and inflammation-driven fibrosis in asthma through the TGF-β1 axis^41^. Such tissues with increased and prolonged EMT in response to chronic damage will increase fibroblast proliferation in healthy tissues^42^. In asthma, it was previously reported that bronchial epithelium differentiation would shift towards the basal epithelial, and those are the most susceptible to TGF-β1-induced EMT, which can give rise to 30–50% of lung fibroblasts in lung fibrosis and allergic asthma^43^.

Increased epithelial expression of MKI67 is linked to increased cycling basal bronchial cells that express higher (MKI67 and TOP2A) to compensate for damaged epithelium^44^. MKI67 suppression in fibroblasts might indicate the loss of proliferative mesenchymal phenotype or population in asthma with specific enrichment in Th2-high phenotype only. Loss of such mesenchymal stem cells can induce lung inflammation and airway remodeling^45^.

The anillin actin-binding protein (ANLN) gene encodes a protein that serves a vital role in cell-cycle progression and controls mitotic progression^46^. ANLN is a crucial mediator of cytokinesis as it binds to actin filaments and cytoskeletal components, serving as a scaffolding protein to coordinate cytokinesis ^47^. When ANLN is nonfunctional, it could disrupt the cellular organization of the bronchial epithelium and predispose it to acute respiratory distress^48^.

Never in Mitosis (NIMA) Related Kinase 2 (NEK2) plays a crucial role in regulating mitotic processes and an essential regulator of the centrosomal cycle besides other diverse cell cycle events ^49–51^. The increased NEK2 kinase levels in bronchial epithelium can increase the inflammatory phenotype of cells as NEK2 was shown to activate the canonical NFκB signaling pathway^52^. On the other hand, the decreased expression of NEK2 in asthmatic fibroblasts can be linked to their resistance to TNFa. NEK2 expression was shown to be suppressed when the TNFa blocker is used in inflammatory diseases^53^. It is recently proved that NEK2 regulates the accumulation of Wnt scaffolding protein Dishevelled (DVL) toward mitosis to remove linker proteins from the centrosome^51^. Its downregulation might derange Wnt signaling and fibroblasts proliferation, as we documented recently ^17^.

Among the three(MKI67, ANLN, and NEK2), MKI67 was shown to be upregulated in Th2 High asthmatic epithelium along with RRM2 and TOP2A, which might be an EMT marker in asthmatic epithelium too.

Our in silico and in vitro results showed that RRM2 was upregulated in asthmatic bronchial epithelium and fibroblasts, specifically in Th2-high asthma. It was previously shown that IL4 could upregulate its expression in resident macrophages ^54^. RRM2 showed differential expression in children with asthma ^55^ and was related to in utero OVA exposure in developing lungs of atopic rats ^56^. Besides M1, RRM2 is the M2 subunit of the ribonucleotide reductase enzyme that regulates the production of 2′-deoxyribonucleoside 5′-triphosphates (dNTP) required for DNA synthesis^57^, and when the RRM2 gene is knocked out, the cell will go to senescence ^58^. RRM2 is only expressed during the late G1/early S phase and degraded in the late S phase to control DNA synthesis and cell proliferation ^59^. RRM2 regulates the expression of the antiapoptotic protein Bcl-2 in apoptosis signaling^60^. Primary epithelial cell maintenance for 3–5 passages can alter the gene expression of NEK2 and RRM2 ^61^. RRM2 is a stress response factor and, if silenced, can inhibit target gene expression in the Wnt/β‑catenin signaling pathway ^62^. It is upregulated in macrophages after smoking^63^. In the context of lung inflammation and repair, when epithelial cells are exposed to LPS, they translocate RRM2 to the nucleus to bind Urokinase-type plasminogen activator (uPA) mRNA 3′UTR and stabilize it to regulate fibrinolysis consequently^64^.

Chromosome untangling during sister chromatids segregation is catalyzed by DNA Topoisomerase II Alpha, the protein encoded by TOP2A ^65^. TOP2A catalyzes topological changes in DNA ^66^ and peaks in the G2/M phase to decatenate DNA during replication ^67^. The expression level of human TOP2A is controlled by its promoter region^68^, and SNP rs11650680 in this promoter was significantly inversely associated with asthma^69, 70^. TOP2 was one of the genes that were changed in offsprings of pregnant mice exposed to TiO2, increasing their susceptibility to asthma^71^.

KRT8, SERPINE1, and ABCA1 were upregulated in asthmatic bronchial epithelium with variable expression in asthmatic bronchial fibroblasts. Of note, their expression was higher in bronchial biopsies than in the brush, indicating a mixed cell origin like mesenchymal Pericytes (SERPINE1) and immune cells (ABCA1). Of note, KRT8 and SERPINE1 were upregulated by oral steroids.

KRT8 It is a known marker of basal luminal precursors cells in the lung ^72^, and such KRT5+, KRT8+ parabasal cell population are the precursors of the luminal cells and act to maintain epithelial homeostasis ^73^. KRT8 previously was shown to be significantly increased in different tissues and secretions of asthmatic patients ^74^. Epithelial KRT8 was shown to be markedly upregulated during viral and nonviral asthma exacerbations in children ^75^.

Plasminogen activator inhibitor‐1 (PAI‐1, also known as SERPINE1) is a serine protease inhibitor that regulates urokinase‐type plasminogen activator (uPA) and tissue‐type plasminogen activator (tPA) activity^76^. Polymorphisms spanning SERPINE1 have shown an association with asthma susceptibility^77^. The SERPINE1 expression is upregulated in response to wounding and, if impaired, can lead to delayed wound healing^78^. SERPINE1 inhibition decreases TGFβ1-induced profibrotic network^79^. SERPINE1 expression was found to be upregulated in hypoxic fibroblasts ^80^, in epithelial cells undergoing EMT, and myofibroblasts at the invasive front^81^. It is a part of the senescence-associated secretory phenotype (SASP) to regulate fibrinolysis and cellular senescence ^82^. SERPINE1 links epithelial cells and inflammatory activity by controlling key inflammatory modulators^83^. LPS induces SERPINE1 secretion, which in turn modulates the immune response to LPS ^84^. Systemic inflammatory TNFα and IL1β can upregulate PAI‐1 synthesis, especially in sepsis‐induced systemic inflammation by gram‐negative bacteremia ^76^. When in contact with virally infected asthmatic bronchial epithelial cells, macrophages can secrete more PAI-1 ^77^.

Our results showed that SERPINE1 expression was more specific to Th2-high asthmatic bronchial epithelium and was upregulated in patients using oral steroids. After the allergen challenge, higher sputum PAI-1 concentration in sputum was documented ^85^. Mast cells in asthmatic airways are a significant source of increased SERPINE1 levels ^86^; these high levels were substantial in tissue repair and remodeling, ultimately affecting asthma severity response to steroids ^87^.

ABCA1 showed higher expression in bronchial biopsies than the pure bronchial epithelium that might indicate the role of infiltrating immune cells or other mesenchymal cells as a source of ABCA1 upregulation. The ABCA1 gene is a member of the ATP-binding cassette family that transports cholesterol and phospholipids across cell membranes. In the lung, ABCA1 regulates reverse cholesterol transport to prevent lipid accumulation and inflammatory cytokines induction ^88^. ABCA1 in alveolar macrophages plays an important role in attenuating the severity of neutrophilic airway inflammation in asthma^89^.

Our results showed that GPRC5A was downregulated in asthmatic bronchial epithelium and upregulated in asthmatic fibroblasts compared to their healthy counterparts. This gene encodes G protein-coupled receptors involved in signaling pathways in development, cellular growth, and epithelial cell differentiation. GPRC5A can block EGFR expression and activation^90^. GPRC5A is expressed in lung type I and type II epithelial cells constitutively^91^. Its deficiency can turn the lung to be more susceptible to endotoxin-induced pulmonary injury^92^ through NFκB and STAT3 signaling pathway^81^, indicating its vital role in lung homeostasis^92^. Cells deficient with GPRC5A produce higher chemokines and cytokines that recruit macrophages through their conditioned medium in an NFκB dependent manner^93^. Repression of GPRC5A is associated with activated IL6 induced STAT3 signaling in tracheal epithelial cells^94^.

Our results showed that Stratifin (SFN) was upregulated in the asthmatic epithelium and was specific to bronchial epithelial cells. SFN, also called 14–3–3 is a p53-inducible gene due to DNA damage to stop cell cycle progression^95^. 14–3–3σ is expressed primarily in epithelial cells as a cell cycle regulator and tumor suppressor^96^. On the other hand, suppression of SFN significantly reduced cell proliferation ^95^. Stratifin plays a significant role in desmosome dynamics in squamous epithelial cells^97^, and epithelial cells with 14–3–3σ loss can undergo epithelial to mesenchymal transition and loss of the epithelial phenotype^98^. SFN can be released from epithelial cells to stimulates matrix metalloproteinase (MMP)-1 expression, and this can affect tissue remodeling^99^. Neighboring epithelial cells releasable 14–3–3σ bind the APN cell surface receptor fibroblasts to act as a potent MMP-1-stimulatory factor ^100^, through c-Fos and c-Jun activity^101^. Such cross-talk should be highly controlled as the ECM continues to accumulate until fibroblasts receive epidermal cells signal to slow down ^102^; any disturbance can lead to excessive fibrosis proving the anti-fibrogenic effect of a controlled release form of stratifin ^103^. SFN was shown to be overexpressed in tissue biopsies of severe equine asthma ^104^.

### In blood

One of the significant goals of asthma research is to identify simple, reliable, and accurate biomarkers that aid in the detection of the disease initiation or progression and lead to a better selection of more precise therapeutic approaches. Serum/blood markers represent the ideal candidate, as they are the least invasive and more patient-friendly approach compared to the bronchial brush or biopsy. Table 4 showed the summary of in silico and patient samples analysis of the ten genes in Blood of asthmatic patients in different settings compared to healthy controls.

**Table 4:**
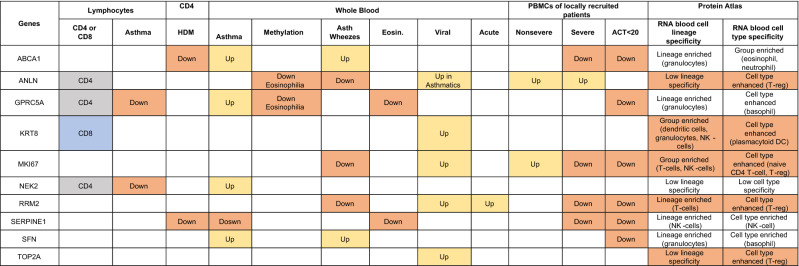
Summary of in silico and patient samples analysis of the ten genes in Blood of asthmatic patients in different settings compared to healthy controls.

Their selective expression on lymphocytes (CD4 vs. CD8), their expression in CD4 cells in response to House Dust Mites (HDM), their expression in asthmatic whole blood (Asth.), their hypomethylation level (Meth) in different phenotypes, in asthmatic with wheezes vs. nonasthmatics wheezers, their expression in eosinophilic asthmatic vs. noneosinophilic, in acute vs. convalescent (acute) and during viral infection and in PBMPs of asthmatic patients from locally recruited cohort compared to healthy controls.

In blood, in silico analysis showed that ABCA1 was higher in asthmatic patient’s whole blood, especially in children with severe wheezes but was downregulated in CD4 lymphocytes stimulated with HDM. ABCA1 expression is specific to eosinophil and neutrophil lineage and is needed for proper Apolipoprotein A-I (apoA-I) mediated reverse cholesterol transport that blocks granulocyte colony-stimulating factor (G-CSF) from initiating neutrophilic inflammation ^89^. ApoA-I mimetic peptides can decrease both neutrophilic and eosinophilic airway inflammation suggesting that the apoA-I/ABCA1 pathway may have a protective effect on asthma^105^.

In our cohort, PBMCs analysis showed that severe asthmatics and patients with ACT < 20 showed downregulation of ABCA1. PBMCs are devoid of eosinophil and neutrophil, so it is expected to have lower ABCA1 in PBMCs than whole blood samples. On the other hand, ABCA1 downregulation might be translated into a loss of ABCA1 anti-neutrophilic effect. Another explanation for such ABCA1 downregulation is the heavy use of steroids in severe asthmatics, as most of these drugs inhibit eosinophils.

Under conditions of excess cellular cholesterol, increases cholesterol efflux through the lipid transporters ATP-binding cassette A1 (ABCA1) is initiated in proliferating T cells to maintain cholesterol homeostasis as they cannot proliferate unless cholesterol satisfies a metabolic checkpoint^106^ that made Abca1A has important anti-inflammatory properties^107^. A significant reduction of ABCA1 mRNA level in leukocytes of patients with atherosclerosis was determined compared with controls^108^. The decreased ABCA1 by HDM might alter T lymphocytes proliferation and function due to deranged cholesterol levels, and that augment the inflammation.

In blood, in silico analysis showed that ANLN mRNA expression was significantly higher in CD4 lymphocytes. ANLN expression is one of the genes shown to be downregulated in T lymphocytes to be Treg cells^109^. ANLN was shown to be upregulated in WBC from mice that developed tolerance to self-antigens (a peptide mimicking anti-DNA IgG sequences containing MHC class I and class II T cell determinant) compared to naive mice^110^. So ANLN upregulation in blood might indicate a decrease of this protective immune cell in response to a stimulus.

In silico analysis showed that ANLN mRNA expression was downregulated in the blood of asthmatic children compared to patients with wheezes, and it was upregulated significantly in asthmatic who develop cold symptoms compared to healthy who developed cold. ANLN mRNA expression was higher in asthmatics (nonsevere and severe) and higher in severe than nonsevere asthmatics PBMCs. Previous reports showed that defective ANLN could result in the abnormal cellular organization of the bronchiolar epithelium, which predisposes to acute respiratory distress^48^.

Our bioinformatics search showed GPCR5A was higher in asthma blood as a whole, while examining lymphocytes fraction only showed GPCR5A downregulation in asthma, indicating that its high level is from cells other than lymphocytes. Interestingly, although GPCR5A expression was lower in eosinophilic patients, it was hypomethylated in the monocytes of those patients, confirming that the main source of GPCR5A expression in blood is not from PBMCs. GPCR5A mRNA expression was significantly down in our cohort of patients in uncontrolled asthma (ACT < 20). It was shown that Gprc5a-ko mice had increased expression of Th2-high biomarkers^111^ and GPCR5A deficiency may lead to impaired pTreg-cell induction in autoimmune diseases as Gprc5a preferentially regulates FoxP3 expression ^112^.

Our in-silico results in asthmatic blood showed that KRT8 mRNA expression was higher in CD8 T lymphocytes in contrast to CD4 T lymphocytes and upregulated significantly in asthmatic who develop cold symptoms due to viral infection. KRT8 showed blood cell lineage of dendritic cells “plasmacytoid DC,” granulocytes, and NK-cells. KRT is considered an anti‐inflammatory gene that is induced by primed monocytes during stress^113^ and increased dramatically in asthmatics who have sensitization and exposure to cockroach allergen ^74^. Some reports showed that the self-reactive immune response to KRT might be an important factor in the inflammatory response^114^.

MKI67 gene expression was downregulated in the blood of asthmatic children’s severe patients with wheezes. Its specific upregulation in response to viral infection can be linked to its T-reg” and NK-cells lineage-specificity. Interleukin-15 was shown to promote the induction of MKI67 on NK cells suggesting a role for NK cells in regulating the immune-mediated pathology in severe asthma^115^. In our cohort, MKI67 was upregulated in the PBMCs of nonsevere but significantly downregulated in severe asthmatic patients and uncontrolled asthma (ACT < 20) compared to healthy controls.

Ki67 was upregulated in CD4+ and CD8+ T cells that had undergone in vitro proliferation after 6-day culture of human whole blood or PBMC with antigens making intracellular Ki67 expression provides a specific, quantitative and reproducible measure of antigen-specific T cell proliferation in vitro, which is a critical function of memory T cells^116^.

Downregulation of MKI67 in immune cells might indicate their exhaustion due to prolonged exposure to antigenic stimulation ^117, 118^. Immune cells with high MKI67 expressed patterns of genes associated with T-cell proliferation and contained a mixture of CD4+ T cells, CD8+ T cells, and Tregs with profound cell proliferation signature that displayed upregulation of pathways associated response to external stimulus pathways^119^. Based on that MKI67 mRNA expression in PBMC can give an accurate impression if the immune cells are in active proliferative phase close to antigen exposure and if the immune cells are impaired like in severe asthma by the disease or in response to immune suppression like steroids.

MKI67 and SERPINE1 expression was higher in nonsevere asthma but was downregulated in severe asthmatics compared to healthy controls. This might indicate their role in the initiation of the disease and then downregulated in severe in response to the therapy or pathophysiological changes, although with the absence of time frame for the patient's asthma progression, such conclusion can be speculative.

Our in-silico results showed that many cell types expressing NEK2 as NEK2 showed Low lineage specificity and low cell type specificity as its mRNA was detected in many blood cells, higher in CD4 T lymphocytes than CD8 T lymphocytes. In CD4 T lymphocytes, only NEK2 was lower in severe asthmatic patients than healthy controls but upregulated in the whole blood of asthmatic, indicating that their source might not be lymphocytes.

The serine/threonine kinase Nek2 is commonly implicated in promoting cell proliferation of B cells leading to an increase in the proportion of immature B cells in the bone marrow with enhanced T cell-dependent immune response^120^. NEK2 is the one most significantly upregulated gene in Multiple Myeloma (MM), a malignancy of terminally differentiated B-cells homing to and expanding in the bone marrow^121^.

Our in-silico analysis showed that RRM2 mRNA expression was upregulated in the blood of an asthmatic patient who had upper respiratory tract infections and those who have acute exacerbation of asthmatic attack but was downregulated in those with severe asthma and wheezes. Protein Atlas data showed that RRM2 is enhanced in T cells, especially Treg. In our cohort, the RRM2 mRNA expression was downregulated in severe asthmatics and those who are not well controlled (ACT < 20). Genetic ablation of RRM2 can induce G1/S phase cell-cycle arrest ^30^ because RRM2 may co-express with relevant genes to regulate the process of lymphocyte infiltration ^122, 123^.

In blood, our in-silico analysis showed that SERPINE1 expression was downregulated in eosinophilic patients or those who were challenged with HDM despite the fact that SERPINE1 local lung expression was upregulated. The SERPINE1 polymorphisms are associated with bronchial reactivity, IgE response, and susceptibility to allergic asthma ^84, 124^. Flagellin was shown to induce SERPINE1 with Th17- and TLR-mediated responses^125^. IL17 producing γδT cells can induce Serpine1 as one of the anti‐angiogenic factors ^126^. On the other hand, full Th17 cell development is dependent on the activity of SERPINE1 ^127^. Th17 cells-derived expression of the PAI-1 along with IL17A was shown to block the anti-inflammatory effect of TGF-β ^83^. In our cohort, SERPINE1 expression in PBMCs was lower in severe asthmatic and those with ACT < 20.

Serine Proteases Inhibitors (serpins) control T lymphocytes functions like the antigen recognition and effector function plus promoting cellular viability and development of long-lived memory T cells through their potent antiapoptotic properties on hematological progenitor cells ^128^. During systemic inflammation, elevated circulating levels of endogenous PAI-1 serve to inhibit IFN-gamma release^129^. Decreased SERPINE1 after HDM in CD4 lymphocytes might indicate a deranged functional T cell.

Our in silico analysis showed that SFN has lineage enrichment for basophil and was upregulated in the whole blood of asthmatics and those with severe asthmatic wheezes. In our cohort, SFN was significantly downregulated in PBMCs with ACT less than 20. Stratifin significantly moderated inflammation by reducing infiltrated CD3(+) immune cells^103^. In patients with chronic rhinosinusitis with nasal polyps, Stratifin was downregulated with dysregulated CD4+ and CD8+ T cells accumulation with impaired suppression of Foxp3+ regulatory T cells leading to a dominant infiltration of lymphocytes and neutrophils and evaluated the inflammatory gene expression^130^. SFN mRNA expression in our cohort was downregulated and might indicate the loss of SFN anti-inflammatory effect leading to inflammatory cells accumulation and impaired regulatory T cells.

TOP2 was upregulated significantly in asthmatic who develop cold symptoms after viral infection linked to their T-reg lineage-specific. During acute lung inflammation, inflammatory macrophages in the airspaces with high proliferative capacity (high TOP2A expression showed a higher inflammatory profile ^131^. TOP2A gene expression was shown previously to be upregulated in human monocytes exposed acutely to Cigarette Smoke ^63^. During chronic inflammatory diseases, TOP2A high expressing cells represent the proliferative subpopulation in exhausted immune cells ^132^.

### Limitation

Because most of the data-driven approach of the manuscript is based on publicly available transcriptomic data, which has significant limitations and drawbacks, a large number of studies using microarrays showed extreme method-to-method variations in the results of the significant differentially expressed genes (DEG) by using different approaches that seek to shorten the list of identified genes ^27^. These limitations affect the proper identification of truly DEG from the equally expressed genes between disease and control ^28^. Age adjustment for the gene expression across different datasets, mainly when the datasets include children while others have adults, is crucial in analyzing omics data and in vivo and in vitro analysis. As per your work, we stressed that as confounding factors that might affect the meaningful interpretations of the biomarker's significance. One of the stringent inclusion criteria that we used to include datasets is that healthy controls and patients groups should be age and sex-matched, making the DEG between different datasets consistent as much as possible. Nevertheless, across other datasets, we might need to add this as a limitation that we did not adjust for age. Also, we tried to use a large number of datasets in different settings using the same analysis method and comparing the normalized gene expression of the identified genes. Another limitation is a relatively small number of cells and patients samples because COVID-19 restricted the patient’s recruitments, although the numbers used were based on the power of calculations to identify minimal numbers needed for meaningful results.

## In conclusion

In conclusion, we used combined in silico, in vitro, and in vivo approaches in an attempt to decipher the molecular basis of asthma heterogeneity by identifying a set of ten genes that are simultaneously differentially expressed in different cells and tissues. We identified factors related to the patients, disease status, and external factors that might deviate the interpretation of the transcriptomic data and proved in vitro and locally recruited patients that the identified genes are biologically crucial to the homeostasis of the lung and in asthma development and progression.

## Supplementary Information

Supplementary Information.
