# Author Correction: Derangement of cell cycle markers in peripheral blood mononuclear cells of asthmatic patients as a reliable biomarker for asthma control

**DOI:** 10.1038/s41598-021-97816-0

**Published:** 2021-09-08

**Authors:** Mahmood Yaseen Hachim, Noha Mousaad Elemam, Rakhee K. Ramakrishnan, Laila Salameh, Ronald Olivenstein, Ibrahim Yaseen Hachim, Thenmozhi Venkatachalam, Bassam Mahboub, Saba Al Heialy, Qutayba Hamid, Rifat Hamoudi

**Affiliations:** 1 College of Medicine, Mohammed Bin Rashid University of Medicine and Health Sciences, Dubai, United Arab Emirates; 2 Center for Genomic Discovery, Mohammed Bin Rashid University of Medicine and Health Sciences, Dubai, United Arab Emirates; 3 Sharjah Institute for Medical Research, College of Medicine, University of Sharjah, Sharjah, United Arab Emirates; 4 Meakins‑Christie Laboratories, McGill University, Montreal, QC Canada; 5 Division of Surgery and Interventional Science, UCL, London, UK

Correction to: *Scientific Reports* https://doi.org/10.1038/s41598-021-91087-5, published online 04 June 2021

The original version of this Article contained errors in the Figure legends of Figure 3 and Figure 4. The legends of these Figures were inadvertently switched.

The legend of Figure 3:

“mRNA expression of (MKI67, RRM2, and TOP2A) genes in bronchial epithelium using GSE67472 dataset to compare healthy controls (n = 43) to Th2-high asthmatics (n = 40) and Th2 low asthmatics (n = 22).”

now reads:

“mRNA expression of the ten genes in bronchial epithelium using, GSE76227 transcriptomic dataset that contains the expression data of 190 bronchial biopsies (BB) and epithelial brushing (BRUSH) from Unbiased BIOmarkers in Prediction of REspiratory Disease outcomes (U-BIOPRED) Project. The normalized gene expression of each of the identified genes was extracted and compared between different subgroups. The datasets were subdivided into nonsevere asthmatic (MAS), severe asthmatics (SAS) (oral steroid naïve (N) vs. oral steroid users (OS).”

The legend of Figure 4:

“mRNA expression of the ten genes in bronchial epithelium using, GSE76227 transcriptomic dataset that contains the expression data of 190 bronchial biopsies (BB) and epithelial brushing (BRUSH) from Unbiased BIOmarkers in Prediction of REspiratory Disease outcomes (U-BIOPRED) Project. The normalized gene expression of each of the identified genes was extracted and compared between different subgroups. The datasets were subdivided into nonsevere asthmatic (MAS), severe asthmatics (SAS) (oral steroid naïve (N) vs. oral steroid users (OS).”

now reads:

“mRNA expression of (MKI67, RRM2, and TOP2A) genes in bronchial epithelium using GSE67472 dataset to compare healthy controls (n = 43) to Th2-high asthmatics (n = 40) and Th2 low asthmatics (n = 22).”

The original Article has been corrected.

