# Supplementary material for: Derangement of cell cycle markers in peripheral blood mononuclear cells of asthmatic patients as a reliable biomarker for asthma control
Source: Sci Rep. 2021 Jun 4;11:11873. doi: 10.1038/s41598-021-91087-5 (PMC8178351; doi:10.1038/s41598-021-91087-5)
Supplement: Supplementary file 1 — Supplementary Information. [file 41598_2021_91087_MOESM1_ESM.docx]

Derangement of Cell Cycle markers in Peripheral Blood Mononuclear cells of asthmatic patients as a reliable biomarker for asthma control

Mahmood Yaseen Hachim^1^, Noha Mousaad Elemam^2^, Rakhee K. Ramakrishnan^2^, Laila Salameh^2^, Ronald Olivenstein^3^, Ibrahim Yaseen Hachim^2^, Thenmozhi Venkatachalam^2^, Bassam Mahboub^2^, Saba Al Heialy^1,3^, Qutayba Hamid^2,3^, and Rifat Hamoudi^2,4^

1. College of Medicine, Mohammed bin Rashid University of Medicine and Health Sciences, Dubai, United Arab Emirates
2. Sharjah Institute for Medical Research, College of Medicine, University of Sharjah, United Arab Emirates
3. Meakins-Christie Laboratories, McGill University, Montreal, QC, Canada
4. Division of Surgery and Interventional Science, UCL, London, United Kingdom

Table S1: List of datasets extracted from GEO omnibus used (check numbers)

| Dataset ID | Tissue/Sample | Study Title | Samples |
| --- | --- | --- | --- |
| GSE67472 | Bronchial Epithelium | Airway epithelial gene expression in asthma versus healthy controls | 105 |
| GSE64913 | Bronchial Epithelium | Altered epithelial gene expression in peripheral airways of severe asthma | 36 |
| GSE76227 | Bronchial Epithelium | Expression data of bronchial biopsies and epithelial brushing from Unbiased Biomarkers in Prediction of REspiratory Disease outcomes (U-BIOPRED) Project | 190 |
| GSE27335 | Bronchial Fibroblasts | Genomic differences distinguish the myofibroblast phenotype of the distal lung from airway fibroblasts | 24 |
| GSE137394 | Whole Blood | Genome-Wide Profiling of Allergic Asthma Peripheral Blood | 309 |
| GSE123750 | Whole Blood | U-BIOPRED blood transcriptomics from children with asthma or wheeze | 216 |
| GSE115823 | Blood | A network of transcriptome modules demonstrates mechanistic pathways of both virus-induced and nonviral asthma exacerbations in children [blood] | 208 |
| GSE69683 | Blood | Expression profiling in blood from subjects with severe asthma, moderate asthma, and nonasthmatics collected in the U-BIOPRED study | 498 |
| GSE31773 | PBMC | Comparison of mRNA expression in circulating T-cells from patients with severe asthma | 40 |
| GSE16032 | PBMC | Gene expression data from severe asthmatic children: PBMC profiles during acute exacerbation versus convalescence | 10 |
| GSE73482 | CD4 | Gene expression patterns in allergen-driven CD4 T cell responses from human atopics with or without asthma. | 144 |
| Total | | | 1780 |

Table S2: The demographic characteristics of the asthmatic patients and control subject’s cohort

| Major Variables | Control | Nonsevere Asthma | Severe Asthma |
| --- | --- | --- | --- |
| **Number of subjects** | 13 | 20 | 20 |
| **Age (Years, SD)** | 34.38±11.26 | 35±13.74 | 49.36±18.40 |
| **Females: Males** | 6: 7 | 5: 15 | 8:12 |
| **BMI** | 24.90±3.961 | 26.94±6.42 | 27.96±5.85 |
| **Childhood Asthma** | 0 (0%) | 15 (75%) | 9 (45%) |
| **Adult Asthma** | 0 (0%) | 5 (25%) | 11 (55%) |
| **Exacerbations/Year** | - | 2.176±3.57 | 7.75±14.72 |
| **Oral Steroid Use per day/Week** | 0 (0%) | 5(25%) | 17(55%) |
| **History of Allergic rhinitis** | 0(0%) | 14 (70%) | 16 (80%) |
| **Peak Flow (l/min)** | 411.81±119.56 | 318.88±71.61 | 292.10±132.43 |
| **ACT Score** | - | 21.1±4.20 | 15.27±6.78 |

Table S3: List of forward and reverse primers for each of the genes assessed by qRT-PCR.

| Gene | Forward Primer | Reverse Primer | Product Size |
| --- | --- | --- | --- |
| 18S rRNA | TGACTCAACACGGGAAACC | TGCCTCCACCAACTAAGAAC | 114 |
| ABCA1 | CCTGCAAAAGGGAGAGAACC | GCAGGTGTTTTGCTTTGCT | 212 |
| ANLN | TTTGTCAGAAATCCGCTTGC | GTGAATGTCAGAGCATCACC | 174 |
| FOSL1 | GCCAAGCATCAACACCAT | CTGATCTGTTCACAAGGCCTTC | 189 |
| GPRC5A | TTTCCCTGTTGGTGATTCTGG | TGAAGGAGGACATGAGGAAGG | 207 |
| JUN | CGGACCTTATGGCTACAGTAAC | CGTTGCTGGACTGGATTATCAG | 188 |
| KRT8 | AGAAGGAGCAGATCAAGACC | GGTTGTTGATGTAGCTCTCG12 | 168 |
| MKI67 | GAAGAGCTCCTAGCAGTCG | GGCCACTTCTTCATTCCAG | 161 |
| NEK2 | TATTGTGAAGGAGGGGATCTGG | CGATGCAATACGGTATGACCAC | 158 |
| RRM2 | GCCATTGAAACGATGCCTTG | GCAAAGGCTACAACACGTTC | 101 |
| SERPINE1(PAI-1) | AACCCAGCAGCAGATTCAAG | CGGAACAGCCTGAAGAAGTG | 195 |
| SFN | CGACAAGAAGCGCATCAT | GTGGTCTTGGCCAGAGAG | 177 |
| TOP2A | GCCCCAAAAGGAACTAAAAGG | GGATTTCTTGCTTGTGACTGC | 165 |


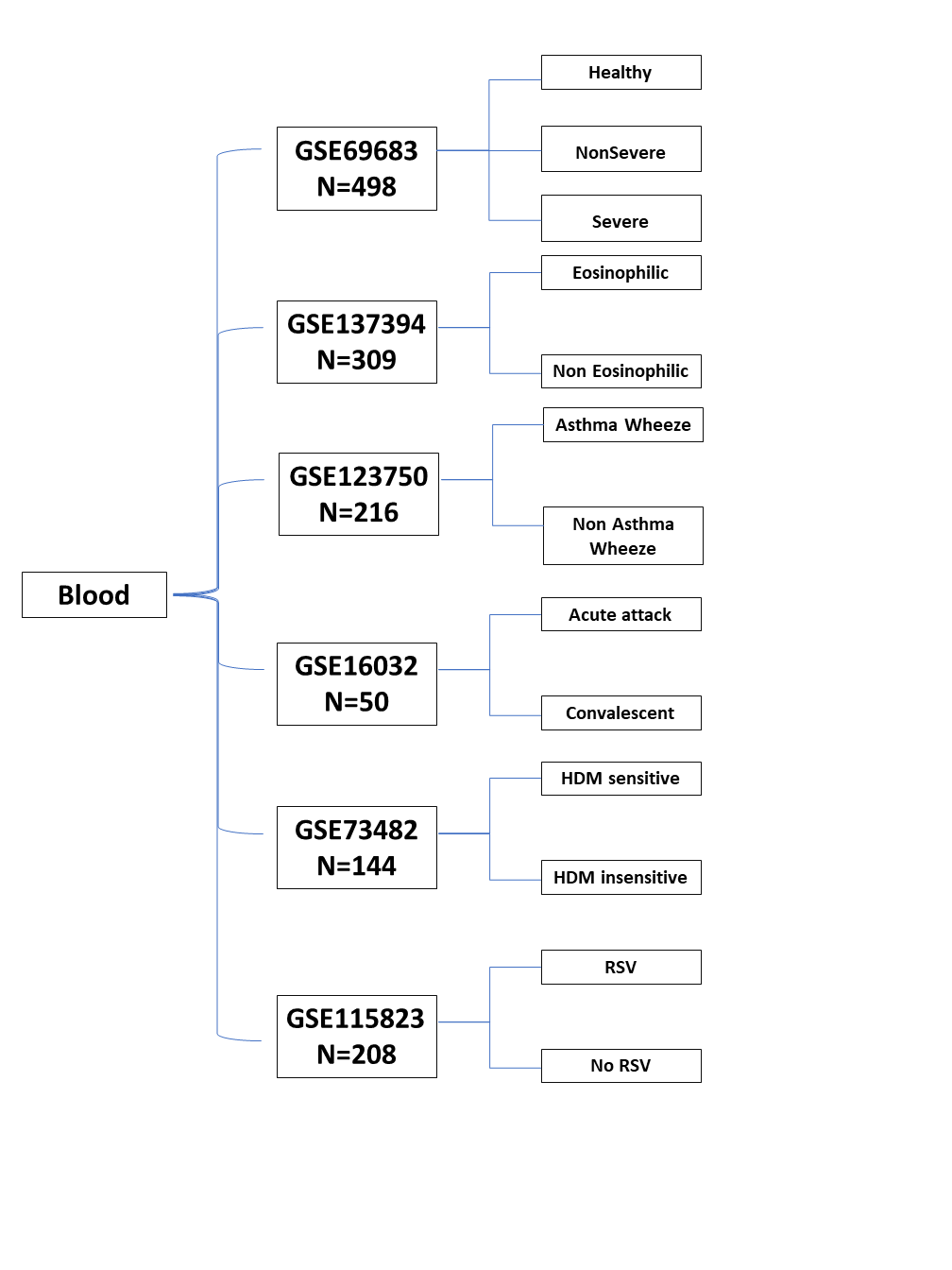


Figure S2

Monocytes of eosinophilic asthmatics showed decreased methylation of GPCR5A and ANLN compared to healthy

GSE59339 dataset was explored to investigate the DNA methylation profiles in peripheral blood monocytes from eosinophilic asthma (EA; n=21), paucigranulocytic asthma (PGA; n=22), neutrophilic asthma (NA; n=9), and healthy controls (n =10). Monocytes of asthmatics especially eosinophilic asthmatics showed decreased methylation of GPCR5A and ANLN compared to healthy, as shown in figure (12). Decreased methylation might be translated into increased mRNA expression of GPCR5A, which was reported to increase the cell number[1]. In macrophages that were differentiated from monocytes using colony-stimulating factor-1 (CSF-1/M-CSF) (CSF-1 Mϕ), there was a selective regulation of GPCR5A and GPCR5B that contributes to skewing toward the M2 macrophage phenotype[2]. Our finding of the decreased methylation of GPCR5A in asthmatics, especially eosinophilic asthmatics, might be explained by increased GPCR5A to be differentiated into M2 macrophage. It was shown that 40-50% of Gprc5a-ko mice developed eosinophilic macrophage pneumonia[3]. ANLN decreased methylation, and increased expression might indicate an increase in cell division as ANLN plays roles during multiple stages of cytokinesis[4].

On the other hand, the KRT8 gene was more methylated in neutrophilic and paucigranulocytic compared to eosinophilic asthmatics (p<0.05) while TOP2A was more methylated in neutrophilic than the paucigranulocytic (p<0.05). These variabilities in gene methylations in different asthma phenotypes can explain the heterogeneity in the interpretation of the biomarkers especially the gene expression markers as many factors can affect their expression.

| 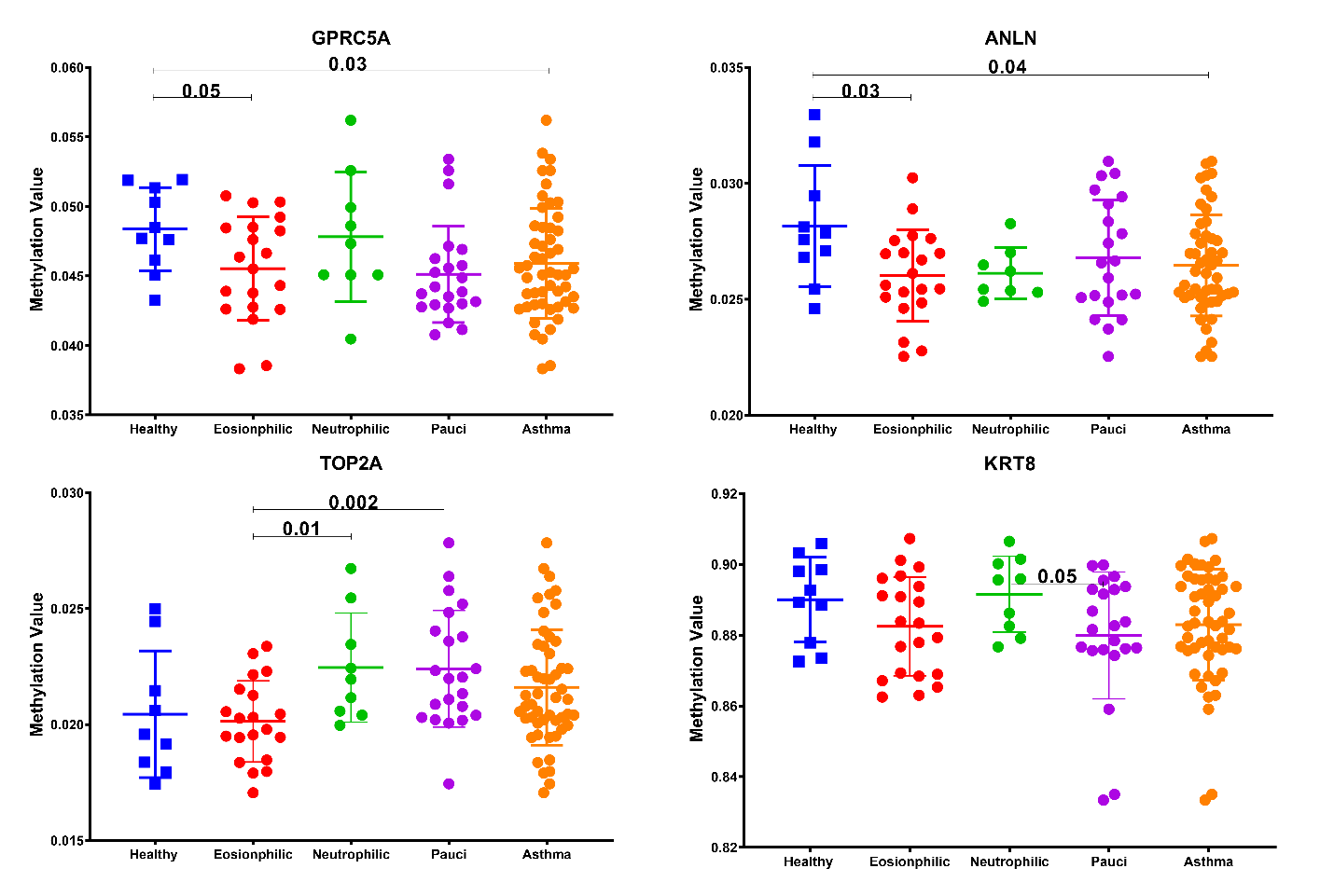 |
| --- |
| Figure S2: DNA methylation value of the 10 genes extracted from GSE59339 dataset showing DNA methylation profiles in peripheral blood monocytes from eosinophilic asthma (n=21), paucigranulocytic asthma (n=22), neutrophilic asthma (n=9), and healthy controls (n =10) |

1. Hirano, M., et al., *Novel reciprocal regulation of cAMP signaling and apoptosis by orphan G-protein-coupled receptor GPRC5A gene expression.* Biochemical and biophysical research communications, 2007. **351**: p. 185-91.

2. Hohenhaus, D.M., et al., *An mRNA atlas of G protein-coupled receptor expression during primary human monocyte/macrophage differentiation and lipopolysaccharide-mediated activation identifies targetable candidate regulators of inflammation.* Immunobiology, 2013. **218**(11): p. 1345-53.

3. Liao, Y., et al., *Gprc5a-deficiency confers susceptibility to endotoxin-induced acute lung injury via NF-κB pathway.* Cell cycle (Georgetown, Tex.), 2015. **14**(9): p. 1403-1412.

4. Zhang, S., et al., *Knockdown of Anillin Actin Binding Protein Blocks Cytokinesis in Hepatocytes and Reduces Liver Tumor Development in Mice Without Affecting Regeneration.* Gastroenterology, 2018. **154**(5): p. 1421-1434.
